# Population Structure, Diversity and Trait Association Analysis in Rice (*Oryza sativa* L.) Germplasm for Early Seedling Vigor (ESV) Using Trait Linked SSR Markers

**DOI:** 10.1371/journal.pone.0152406

**Published:** 2016-03-31

**Authors:** Annamalai Anandan, Mahender Anumalla, Sharat Kumar Pradhan, Jauhar Ali

**Affiliations:** 1 Division of Crop Improvement, National Rice Research Institute, Cuttack, Odisha, India; 2 Plant Breeding, Genetics and Biotechnology Division, International Rice Research Institute, Los Baños, Philippines; Institute of Crop Sciences, CHINA

## Abstract

Early seedling vigor (ESV) is the essential trait for direct seeded rice to dominate and smother the weed growth. In this regard, 629 rice genotypes were studied for their morphological and physiological responses in the field under direct seeded aerobic situation on 14^th^, 28^th^ and 56^th^ days after sowing (DAS). It was determined that the early observations taken on 14^th^ and 28^th^ DAS were reliable estimators to study ESV as compared to56^th^ DAS. Further, 96 were selected from 629 genotypes by principal component (PCA) and discriminate function analyses. The selected genotypes were subjected to decipher the pattern of genetic diversity in terms of both phenotypic and genotypic by using ESV QTL linked simple sequence repeat (SSR) markers. To assess the genetic structure, model and distance based approaches were used. Genotyping of 96 rice lines using 39 polymorphic SSRs produced a total of 128 alleles with the phenotypic information content (PIC) value of 0.24. The model based population structure approach grouped the accession into two distinct populations, whereas unrooted tree grouped the genotypes into three clusters. Both model based and structure based approach had clearly distinguished the early vigor genotypes from non-early vigor genotypes. Association analysis revealed that 16 and 10 SSRs showed significant association with ESV traits by general linear model (GLM) and mixed linear model (MLM) approaches respectively. Marker alleles on chromosome 2 were associated with shoot dry weight on 28 DAS, vigor index on 14 and 28 DAS. Improvement in the rate of seedling growth will be useful for identifying rice genotypes acquiescent to direct seeded conditions through marker-assisted selection.

## Introduction

Rice (*Oryza sativa* L.) is widely cultivated under irrigated and rainfed conditions, with cultivated varieties and landraces that specifically adapt to these situations. In India, more than 50% of rice areas under rainfed conditions are cultivated as direct seeded rice (DSR). The existing rainfed upland varieties are of short duration type, drought tolerant and low yielding and possess early vigor trait. Direct seeding is also a common practice in low land rice of India and weed is the major problem in those situations. Early seedling vigor (ESV) trait of rice will help in competing with weed and this trait is needed in lowland rice. While, irrigated rice varieties are high yielding and relatively poorer in early vigor as compared to rainfed varieties. In rice growing areas of South and South East Asia, ESV trait has been exploited in rainfed upland cultivar development but not to the same extent in rainfed low land and irrigated situations, mainly due to the narrow genetic base of selecting attributes primarily for higher grain yields. Attempts were made to introgress ESV from upland landraces to high yielding cultivars; however, significant progress could not be achieved due to complex nature of the trait [1]. Further, only a small fraction of available landraces has been used in practical breeding. Traditional native landraces are widely grown under rainfed lowland conditions by resource poor farmers in India due to lack of suitable improved varieties for such harsh conditions. Most of these landraces are less productive, but they possess weed-smothering ability by early vigor and excellent growth adaptation features to direct seeded conditions. Due to unpredictable rainfall patterns, direct seeded rice has become regular practice under rainfed situation. Conversely, due to labor scarcity and unavailability of timely irrigation water, the farmers in the irrigated ecosystems are also adapting to dry direct seeding techniques. However, currently available modern rice varietal architecture with semi-dwarf stature and reduced seedling vigor is not amenable to dry direct seeded conditions [1]. It is clear that, ESV is relatively restricted to upland varieties as compared to transplanted rice varieties. Therefore, for DSR ESV is an important trait to achieve higher biomass, smothering weed ability with higher grain yields [2].

For better understanding of ESV trait, it is important to know the genetic relationship among a large set of cultivated genotypes by using DNA markers efficiently. Among the different DNA markers, simple sequence repeats (SSR) are most commonly used, as they are hyper variable, co-dominant, robust, multi-allelic in nature, chromosome specific and greatly facilitated linkage map construction and use in plant breeding. SSR markers are widely employed to assess genetic diversity and genetic structure in rice and several other crops. However, limited information is available on classification of ESV in rice cultivars using DNA markers. Molecular markers-based genetic maps facilitate applied genetics and breeding program [3].

ESV and its component traits being complex quantitative nature, their involvement with several physiological processes and influences of G x E interactions makes these traits to achieve slower progress for genetic enhancement. Therefore, integration of marker tools into breeding schemes provides an opportunity to handle such challenges and facilitates marker-assisted selection. Continuous variation could be observed for ESV within a segregating population and frequency of favorable alleles at many loci could also be achieved by selection. QTL mapping based on segregating population helps to identify loci for quantitative and qualitative traits with limited number of segregating alleles at any locus and low resolution typically in the range of 10-30cM [4]. To overcome such situations, association mapping based on the linkage disequilibrium is popularly adapted to achieve a higher resolution and targets multiple alleles at individual loci by exploiting historical recombination events available in germplasm and by identifying association between marker and phenotype of larger number of traits. Till date, very few researches on association studies in rice for ESV have been done. Therefore, the present study examined to explore; i) the characteristic differences for ESV of 629 eco-geographical rice genotypes, ii) genetic diversity of the selected genotypes using molecular markers, and iii) genomic regions associated with ESV related traits.

## Materials and Methods

### Experiment 1

**Fig 1:**
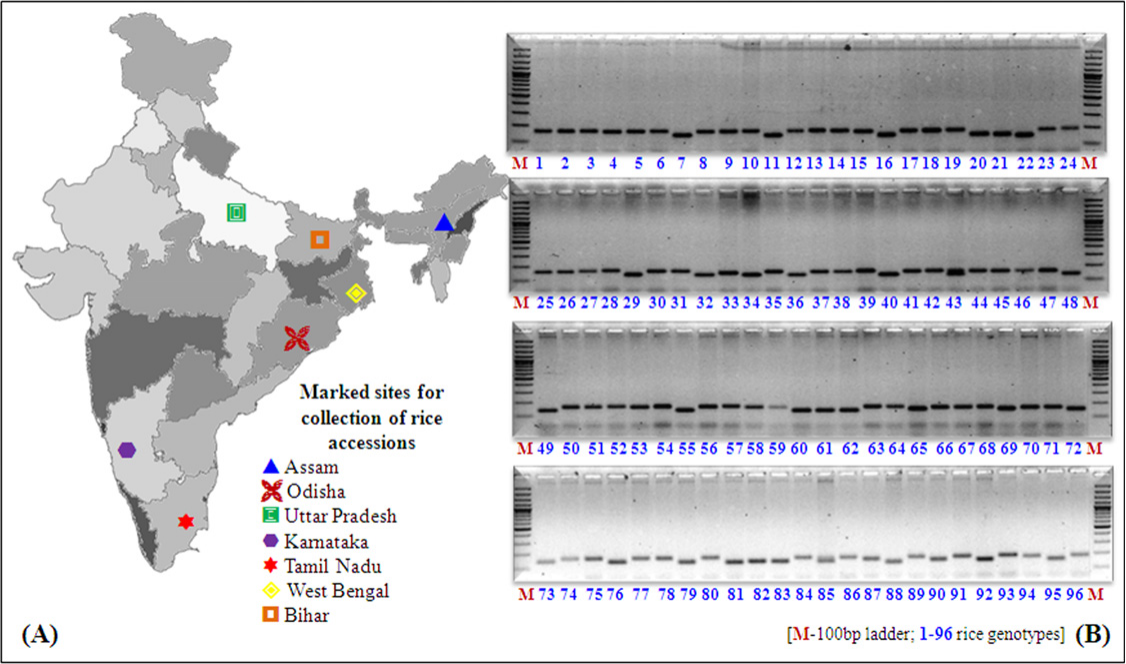
(A) Diagrammatic representation of collection of rice germplasm from different states of India; (B) Molecular profiling of mini-core 96 rice accessions with polymorphic microsatellite markers.

The total of 629 rice (*Oryza sativa*. L) genotypes (Fig 1) were representative of breeding lines and landraces, comprising of 25 tropical *japonica*, 57*indica* landraces, 127 breeding lines, and 427 ARC (Assam Rice Collection) accessions cited in S1 Table. The seeds were obtained from Gene bank of National Rice Research Institute (NRRI), Cuttack, India. The experiment was conducted at NRRI during 2013 dry season (experiment 1) in upland situation (20°.48’N, 85°.86’E). The experimental site soil had 34.4% sand, 29.1% silt, 32.4% clay with bulk density of 1.46 Mg/m^3^. Seeds of each of the genotype were direct sown in 4 m long row with 15 cm apart and 20 cm between rows in augumented block design. Pre-emergence herbicide Pendimethalin was sprayed at the recommended rate within 24 hr of irrigation and maintained weed-free through the growing period by hand weeding. Crop was raised with recommended fertilizers doses of 80 kg nitrogen ha^-1^, 40 kg phosphorus ha^-1^, and 40 kg potash ha^-1^. The field was maintained under non-saturated aerobic condition with supplemental surface irrigation.

In order to study the variability among genotypes for ESV, several vegetative traits were observed. Fourteen days after sowing (DAS), six rice plants in the middle row from each genotype were selected to study the shoot length (cm), number of leaves, root length (cm) and shoot dry weight (DW) (g). The same set of traits was observed on 28 and 56 DAS with number of tillers per plant and leaves per tiller. Growth rate of seedlings were measured to assess ESV by vigor index (VI) as suggested by Maguire [5].

$$\text{VI}=\frac{\text{Rate of ger min ation}\;(\%)}{\text{Seedling dry weight}\;(\text{mg})}$$

While, germination rate (GR) was calculated as

$$\text{GR}=\frac{{\text{X}}_{1}}{{\text{Y}}_{1}}+\frac{\left({\text{X}}_{2}-{\text{X}}_{1}\right)}{{\text{Y}}_{2}}+\ldots +\frac{\left({\text{X}}_{\text{n}}-{\text{X}}_{\text{n}-1}\right)}{{\text{Y}}_{\text{n}}}$$

Where, X_n_ is the number of germinated seeds on the n^th^ day and Y_n_ is the number of days from the first experimental day.

Absolute growth rate (AGR) was calculated as

$$\text{AGR}=\frac{\left({\text{h}}_{2}-{\text{h}}_{1}\right)}{\left({\text{t}}_{2}-{\text{t}}_{1}\right)}{\text{cm day}}^{-1}$$

Where, h_1_ and h_2_ are the plant height at times t_1_ and t_2_ respectively.

Relative growth rate (RGR) was determined by measuring plant dry weight periodically during growth and is represented as mg g^-1^day^-1^.

$$\text{RGR}=\frac{\left({\text{log}}_{\text{e}}\;{\text{W}}_{2}-{\text{log}}_{\text{e}}\;{\text{W}}_{1}\right)}{\left({\text{t}}_{2}-{\text{t}}_{1}\right)}$$

Where, W_1_ and W_2_ are the plant dry weights at times t_1_ and t_2_ respectively.

Crop growth rate (CGR) measured the plant dry weight of a particular ground area at a regular interval of time divided by land area and represented as g m^-2^ day^-1^.

$$\text{CGR}=\frac{\left({\text{W}}_{2}-{\text{W}}_{1}\right)}{\text{P}({\text{t}}_{2}-{\text{t}}_{1})}$$

Where w_1_ and w_2_ are the plant dry weights at times t_1_ and t_2_ respectively, P = spacing (m^2^).

An initial descriptive statistics, including mean, standard deviation, minimum and maximum values, coefficient of variation (%), skewness, kurtosis, and distribution pattern was performed by box plot technique. PCA was performed to estimate Euclidean distance between two genotypes in the multivariate space and adaptation of genotypes to specific environment [6–7]. These analyses were performed using the Windostat 7.5 software (Indostat Services, Hyderabad, India.). Discriminate function analysis was applied to find the best variable(s) that can discriminate high and low seedling vigor genotypes. For this, the genotypes were divided arbitrarily into three groups based on the shoot length and dry weight (Tables 1 and 2).

**Table 1:** Arbitrary classification of seedling vigor on 14^th^ and 28^th^ days after sowing.

S. no	Shoot length (cm)	Categories	Shoot weight(g)	Categories
1	0–15	Short	<0.04	Low
2	16–30	Intermediate	0.05–0.1	Intermediate
3	>30	Tall	>0.1	High

**Table 2:** Arbitrary classification of seedling vigor on 56^th^ DAS.

S. NO	Shoot length (cm)	Categories	Shoot weight(g)	Categories
1	0–30	Short	<2.9	Low
2	31-55-	Intermediate	3–6.00	Intermediate
3	>55	Tall	>6.1	High

Based on the broad trend observed in the PCA and phenotypic data. Forward stepwise discriminate function analysis (DFA) was carried out to understand the combination of variables, which could best explain the grouping in STATISTICA ver.10 (Stat soft, Inc, Tulsa, ok, USA). In DFA, a larger eigen value indicates that the discriminant function is more useful in distinguishing between the groups.

### Experiment 2

Ninety-six genotypes were selected representing high, medium and low vigor genotypes with almost equal proportions from each group were selected for the panel from the results of the experiment 1. Groups were defined by following the scale presented in Table 1. The selected genotypes were raised in earthen pots (20 cm height and 15 cm diameter) containing upland soil and farmyard manure in 3:1 ratio in three replications during 2014 dry season. Three plants were maintained per pot. The same set of 96 genotypes was raised on upland situation in augumented design with similar agronomic practices in four-meter row length as followed in experiment 1. Five plants from each genotype were selected for measurement of shoot length (cm), root length (cm), leaf length (cm), leaf width (cm), number of leaves, shoot dry weight (g), root dry weight (g), and VI on 14^th^ and 28^th^ DAS. By using these variables, RGR, AGR and CGR were derived to measure growth rate of seedlings as mentioned in experiment 1. PCA based clustering was performed to find out the pattern of phenotypic diversity and variables contributions to diversity using the Windostat 7.5 software.

#### DNA isolation and selection of SSR markers

The experimental material comprised of selected 96 genotypes that were direct sown in the pot and grown in net house at NRRI, Cuttack. After 20 days of sowing, young leaves were harvested and genomic DNA was isolated from the bulked leaf sample following CTAB method [8]. The quantity was estimated by spectrophotometer and agarose gel electrophoresis using lambda DNA of known concentration. The isolated DNA samples were diluted in T_10_E_1_ buffer (10 mM Tris-HCl, 1 mM EDTA, pH 8.0) to obtain the final concentration of 15 ng/μl for amplification.

A *priori* information was used to select ESV linked 52 SSR markers from the reports of earlier mapping population studies and the distribution of markers covered all the chromosomes to illustrate the diversity [1].

#### PCR amplification and electrophoresis

The PCR amplification was executed with 20 μl of PCR mixture contained 30 ng of genomic DNA, 1x PCR buffer, 200 μM dNTP mix, 4 picomoles of each forward and reverse primers, 2 mM of MgCl_2,_ and 1U of Taq DNA polymerase (MBI, Fermentas, USA). Template DNA initially denatured at 94^°^C for 5 min followed by 36 cycles of denaturation at 94^°^C for 1 min, annealing at 55–67^°^C (varies upon primers) for 1 min and extension at 72^°^C for 5 min followed by final extension at 72^°^C for 10 min. The amplification products were separated on 2.5% agarose gel containing ethidium bromide using 1x TBE buffer. DNA fragments were visualized under UV transillumination using Alpha Innotech gel documentation system (Flour Chem^TM^ 5500, Alpha Innotech, USA).

#### Data analysis

Amplicons were scored according to their product size for each genotype and primer combination. Number of alleles (N), major allele frequency (A), observed heterozygosity (H_o_), expected heterozygosity (He), and PIC, for each SSR locus were determined by using Power marker 3.25[9]. A PIC value of each marker was determined as suggested by Botstein et al. [10].

Further, the allelic data were subjected to estimation of genetic distances among genotypes using simple matching coefficients by bootstrapping 10,000 times and they were clustered using neighbor joining method [11]. PCoA was performed to highlight the resolving power of the ordination and the first two components were used to represent the genotypes in the graphical form. PCoA and dissimilarity matrix were performed by using DARwin software version 5.0 [12]. Further, Analysis of molecular variance (AMOVA) was performed to describe variance components among individuals and the population differentiation among the seven assumed sub populations using GeneAlEx 6.41 program [13] with 1000 permutations. Genetic differentiation among the assumed sub population was analyzed using Nei’s gene diversity statistics [14] using POPGENE program version 1.31 [15]. To identify the genetic structure of given population and assign individuals to populations, the software STRUCTURE version 2.3.3 [16] were used. To derive the optimal number of groups (K), STRUCTURE was run with K varying from 1 to 10, with five runs for each K value. To determine the true value of K, *ad hoc* statistic ΔK was followed [17]. Parameters were set to 1,00,000 burn-in periods and 10,000 Markov Chain Monte Carlo (MCMC) replications after burn-in with an admixture and allele frequencies correlated model.

#### Association analysis

To find the genetic relatedness between phenotypic performances of the rice accessions and SSR makers, two statistical models were used; i) General linear model (GLM) and ii) Mixed linear model (MLM) in TASSEL version 5.0[18]. Significantly associated markers with traits were identified on the traits of their R^2^ and *p*-value.

## Results

### Experiment 1

#### Variation in ESV related morphological traits on 14, 28and 56 days after sowing

In the present investigation, distribution pattern of ESV traits of 629 genotypes were presented in S1A–S1H Fig. Among the ESV traits studied on 14^th^, 28^th^ and 56^th^ days after sowing (DAS), number of leaves and germination rate were negatively skewed (S <-1) on 14 DAS (S1 Fig), while dry weight (DW), vigor index (VI) and crop growth rate (CGR) were positively skewed (S>1) on 56 DAS. Normal distributions were observed for all other traits. Visible trait differences were observed among the genotypes studied. Among the ESV traits studied at different stages, vigor index on 56 DAS showed the largest relative difference with 40.75 fold followed by DW on 56 DAS (37.40 fold) and CGR between 28 and 56 DAS (37.40 fold). The higher level of difference could provide an opportunity to select genotypes with ESV. Inter-quartile range (IQR) is a measure of variability. Among all the traits studied, lower IQR was observed for DW on 14 DAS and RGR between 28 and 56 DAS followed by VI on 14 DAS. Germination rate and number of leaves/tiller on 56 DAS have significant number of outliers. Among the traits studied on 14 DAS, shoot length varied from 9.4to 26.6cm as measured in ‘Sharvesh’(9.40 cm) and ‘ARC 10797’ (26.58 cm)respectively with mean of 16.75 cm and ‘ARC 10797’ exhibited 1.58 times higher than the mean value. Coefficients of variation (CV) indicating variability among the genotypes were high for VI on 56 DAS (57.93%) followed by CGR between 28^th^ and 56^th^ (52.14%), DW on 56 DAS (51.44%) and VI on 28 DAS (42.23%). Higher CV suggests that, the selected material in this experiment exhibited higher variability.

#### Grouping pattern of genotypes and association between variables

**Fig 2:**
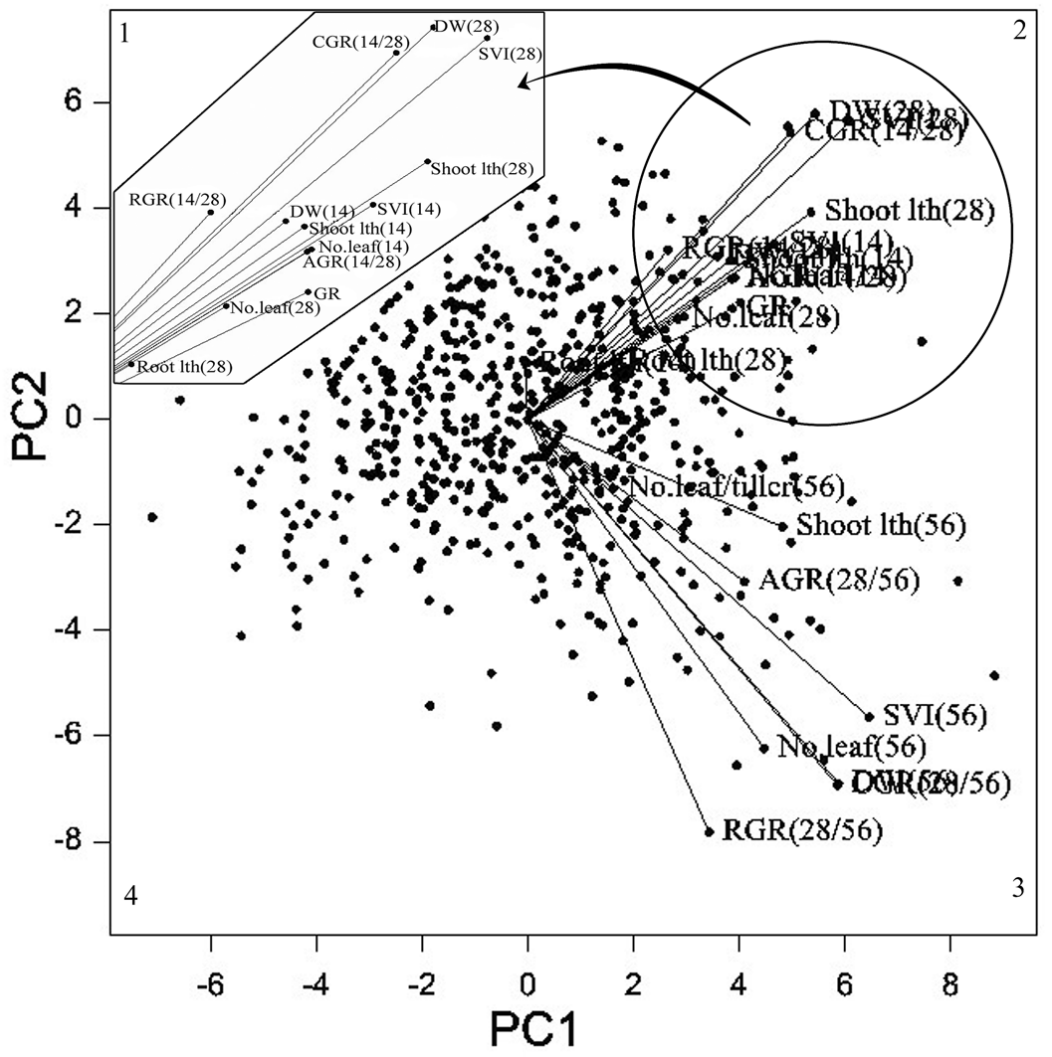
Spatial distribution of 629 rice genotypes for early seedling vigor on 14, 28 and 56 days after sowing for the first two principal components.

Principal component analysis (PCA) was performed to identify association between traits, responsible traits for ESV and grouping pattern of rice genotypes on the basis of the traits. The first two PCs accounted for about 71.47% of the total variability with >1.0 eigen value. The biplot in Fig 2 shows a strong relationship between the DW and VI (r = >0.900), dry weight and shoot length (r = >0.400) and VI and CGR (r = >0.800) irrespective of different days of observation (14, 28 and 56 DAS). Cultivar-by-trait biplot indicated that the traits were grouped into two, based on days of observation. Traits observed on 14 and 28 DAS, were grouped together and separated from traits observed on 56 DAS (Fig 2). Genotypes with early vigor and growth on 14 and 28 DAS were grouped together in quadrant 2, whereas genotypes with high biomass on 56 DAS were grouped in quadrant 3. The discriminate function is statistically significant with high canonical correlation (r = 0.8398). Further, it discriminated high and low seedling vigor genotypes and found that shoot length on 28 DAS showed the lowest partial lambda, the highest F-remove value and standardized coefficient, followed by shoot weight on 28 DAS.

### Experiment 2

#### Traits and grouping pattern of selected genotypes

**Fig 3:**
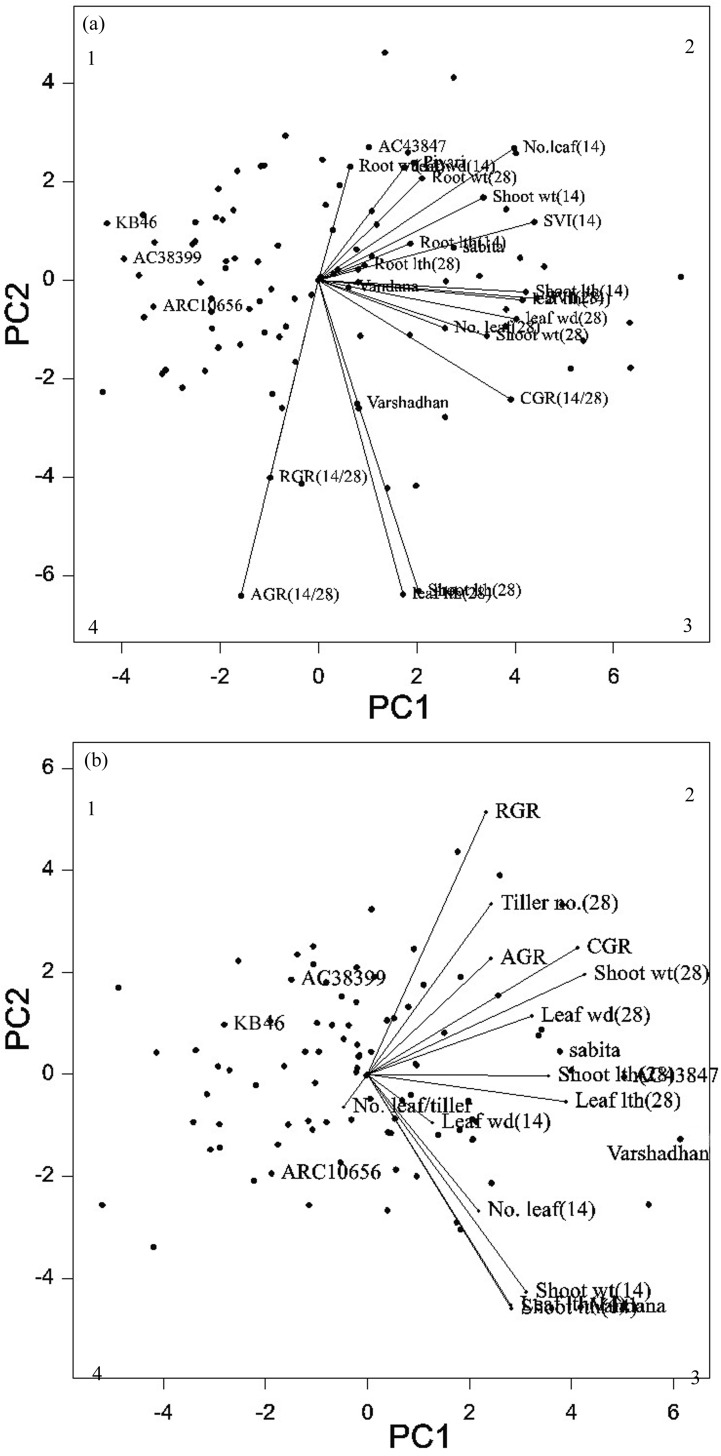
(a, b). Spatial distribution of selected 96 rice genotypes for early seedling vigor on 14 and 28 days after sowing for the first two principal components. (a) Net house and (b) Field conditions.

The selected 96 genotypes were raised under net house and field condition during dry season. PCA was executed for all the 96 genotypes under both situations to identify trends among low and high early seedling vigor genotypes and the traits responsible for source of the variability (Fig 3A and 3B). The PC_1_ accounted for about 60.71% and PC_2_ accounted for 21.22% of the total variance (totaling 81.93%) under net house condition. From Fig 3A, it is inferred that the longest vector loading such as AGR, leaf length and shoot length on 28 DAS are the major discriminators. However, under field condition RGR, leaf length and shoot length on 14 DAS are the major discriminators (Fig 3B). In PCA under field condition, first and second component explained 75.08% and 15.88% (totaling 90.96%) of the variance respectively. The PCA under both conditions, groups the genotypes into two groups early vigor genotypes on right side and non-early vigor genotypes on left side.

#### SSR marker segregation

In the present experiment, 96 rice genotypes include 85 landraces and 11 modern/breeding lines representing six major rice growing states of India. Initially the experiment was begun with 52 SSR markers to assess the genetic relatedness. Among them, 39 markers generated polymorphic bands (Table 3). The number of alleles amplified per marker varied from 2 to 7 with an average of 3.28 per locus in 96 genotypes. PIC values ranged from 0.04 (RM3839) to 0.37 (RM148) with a mean of 0.24. Observed heterozygosity (H_o_) ranged from 0.04(RM3839) to 0.97 (RM148) with an average heterozygosity across all 39 loci was 0.42. None of the loci exhibited heterozygosity as zero. The expected heterozygosity (gene diversity) (H_e_) ranged from 0.04 to 0.50 with an average of 0.30. Major allele frequency was also calculated for all 39 loci, which ranged from 0.52 to 0.98 with an average of 0.79 (Table 4).

**Table 3:** List of early seedling vigor (ESV) traits associated polymorphic microsatellite markers in rice (1).

S.No	Marker	Traits associated	Chr	Amplicon Size (bp)	Tm	Reference
1	RM13	SL,RL,DW,GR,SDW	5	141	55	[19]
2	RM125	GR, GP and GI	7	127	55	[20]
3	RM26	TDW,SDW,RDW,VI,RARL,SL,GR	5	112	55	[21–24]
4	RM87	SDW	5	151	55	[19,21]
5	RM3839	SDW	4	218	50	[25]
6	RM161	RL,DW,SEV,FV.GR	5	187	61	[26]
7	RM218	SL,FV	3	148	55	[27]
8	RM228	SS,SL,FW,WS	10	154	55	[26–27]
9	RM9	GR,SDW,SFW	1	136	55	[20]
10	RM6091	GR,GI	11	126	50	[20]
11	RM7075	GR	1	155	50	[20]
12	RM258	RDW,	10	148	55	[21]
13	RM3428	GP,GI,NL,SS	11	156	55	[20]
14	RM334	SDW,	5	182	55	[19, 21]
15	RM148	GR,SL,SEV,FW	3	129	55	[19, 26, 27]
16	RM250	SDW,	2	153	55	[28]
17	RM221	SEV,	2	192	55	[26]
18	RM264	GR,SL,RL	8	178	55	[19]
19	RM230	GR,SL,RL	8	257	55	[19]
20	RM253	SL, GR,	6	141	55	[19, 23]
21	RM85	GR,SL,RL,SEV,FW	3	107	55	[19, 26]
22	RM340	SL,GP	6	163	55	[19, 20]
23	RM249	SV, FV,	5	121	55	[19]
24	RM225	SV,GR, SDW, SL	6	140	55	[19, 29]
25	RM263	SL,SDW	2	199	55	[22]
26	RM30	GR, SDW, SL	6	105	55	[29]
27	RM223	SW,GR,SL,SEV	8	165	55	[19, 26]
28	RM1339	SL	1	144	55	[30]
29	RM16	SL,SDW,RL,GR	3	181	55	[19]
30	RM252	SEV,GP,GR,RA	5	216	55	[19, 21, 26]
31	RM336	GR, SDW, SL	7	154	55	[29]
32	RM8085	SL,	1	126	55	[30]
33	RM19	GR, SDW, SL	12	226	55	[29]
34	RM6	SDW	2	163	55	[22]
35	RM168	SL,DW,GR,RL,SEV,FW	3	116	55	[19, 26]
36	RM224	GR, GR, SDW, SL	11	157	55	[29, 31]
37	RM7389	SL	3	111	55	[30]
38	RM21	GR, SDW, SL	11	157	55	[29]
39	RM341	GR,	2	172	55	[23]

SDW-Shoot dry weight; SL-Shoot length; RDW-Root dry weight; RL-Root length; TFW-Total fresh weight; TDW-Total dry weight; RA-Root activity; GP-Germination percentage; GR-Germination rate; GI-Germination index; FGC-First germination count; EGC-Ending germination count; ME-Mesocotyl elongation; PL-Plumule length; COL-Coleorhiza length; CL-Coleoptile length; VI-Vigor index; RSC-Reducing sugar content; SW-Seed weight, SE-Seedling establishment, SFW-Seedling fresh weight, SS-Seed size, SEV-Seedling early vigor, LA-Leaf area, NL-Number of leafs; SR-Seed reserve utilization efficiency, WS-Weight of mobilized seed reserve, FV-Field vigor, SV-Seed vigor.

**Table 4:** List of microsatellite markers used for genotyping rice accessions for early seedling vigor traits along with their genetic diversity parameters.

S.No	Marker	Chromosome	Molecular Weight	Gene diversity	Heterozygosity	PIC	Allele frequency	Total allele	Unique allele	Rare allele
1	RM125	7	127	0.10	0.10	0.09	0.95	3	0	1
2	RM13	5	141	0.14	0.15	0.13	0.93	3	0	0
3	RM1339	1	144	0.30	0.36	0.25	0.82	5	0	2
4	RM148	3	129	0.50	0.97	0.37	0.52	3	0	0
5	RM16	3	181	0.30	0.38	0.26	0.81	6	0	0
6	RM161	5	187	0.30	0.36	0.25	0.82	3	0	0
7	RM168	3	116	0.27	0.32	0.23	0.84	2	0	0
8	RM19	12	226	0.47	0.75	0.36	0.63	6	0	0
9	RM21	11	157	0.38	0.50	0.30	0.75	3	0	0
10	RM218	3	148	0.46	0.71	0.35	0.65	3	0	0
11	RM221	2	192	0.49	0.86	0.37	0.57	3	0	0
12	RM223	8	165	0.11	0.11	0.10	0.94	3	0	1
13	RM224	11	157	0.44	0.66	0.34	0.67	3	0	1
14	RM225	6	140	0.40	0.56	0.32	0.72	2	0	0
15	RM228	10	154	0.12	0.13	0.11	0.94	2	0	0
16	RM230	8	257	0.12	0.13	0.11	0.94	3	0	0
17	RM249	5	121	0.42	0.60	0.33	0.70	2	0	0
18	RM250	2	153	0.11	0.11	0.10	0.94	3	0	0
19	RM252	5	216	0.44	0.67	0.35	0.67	4	0	0
20	RM253	6	141	0.14	0.16	0.13	0.92	2	0	0
21	RM258	10	148	0.39	0.53	0.31	0.73	5	0	0
22	RM26	5	112	0.45	0.68	0.35	0.66	4	1	1
23	RM263	2	199	0.35	0.45	0.29	0.78	3	0	0
24	RM264	8	178	0.45	0.70	0.35	0.65	3	0	0
25	RM30	6	105	0.47	0.76	0.36	0.62	2	0	0
26	RM334	5	182	0.38	0.51	0.31	0.74	4	0	1
27	RM336	7	154	0.48	0.82	0.37	0.59	2	0	0
28	RM340	6	163	0.13	0.14	0.12	0.93	5	0	1
29	RM341	2	172	0.46	0.73	0.36	0.64	4	0	0
30	RM3428	11	156	0.13	0.14	0.12	0.93	2	0	0
31	RM3839	4	218	0.04	0.04	0.04	0.98	5	0	1
32	RM6	2	163	0.45	0.68	0.35	0.66	3	0	0
33	RM6091	11	126	0.28	0.34	0.24	0.83	2	0	0
34	RM7075	1	155	0.16	0.18	0.15	0.91	4	0	0
35	RM7389	3	111	0.29	0.35	0.25	0.82	2	0	0
36	RM8085	1	126	0.14	0.16	0.13	0.92	2	0	0
37	RM85	3	107	0.25	0.29	0.22	0.85	2	0	0
38	RM87	5	151	0.11	0.11	0.10	0.94	3	0	0
39	RM9	1	136	0.23	0.27	0.21	0.86	7	0	0
	**Mean**			**0.30**	**0.42**	**0.24**	**0.79**	**3.28**	**0.03**	**0.23**

#### Genetic relatedness by cluster and principal coordinate analysis (PCoA)

**Fig 4:**
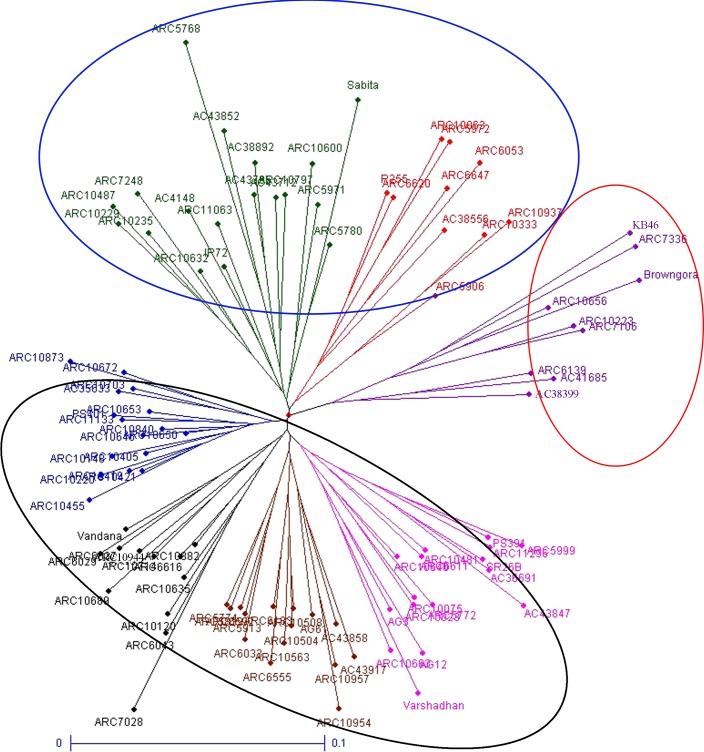
Unrooted neighbor joining tree constructed for 96 rice genotypes based on trait linked SSRs.

Cluster analysis was carried out to assess genetic distance and the dissimilarity matrix-using neighbor joining method. In the Unrooted tree genotypes were grouped into three major clusters (Fig 4). Cluster 1 has 59 genotypes, they sub grouped into cluster 1a and cluster 1b with 43 and 16 genotypes respectively. Similarly, cluster 2 was sub grouped into cluster 2a that contains 18 genotypes and cluster 2b with 10 genotypes. Cluster 3 was the smallest containing 9 genotypes. On the basis of geographical location, no significant grouping was observed. However, sub group 1b and 2b and cluster 3 having genotypes of landraces alone. Further, sub group 1a and 2a clustered genotypes of both improved and landraces. The genotypes of tropical *japonica* were grouped into two sub groups as 2a and 2b.

**Fig 5:**
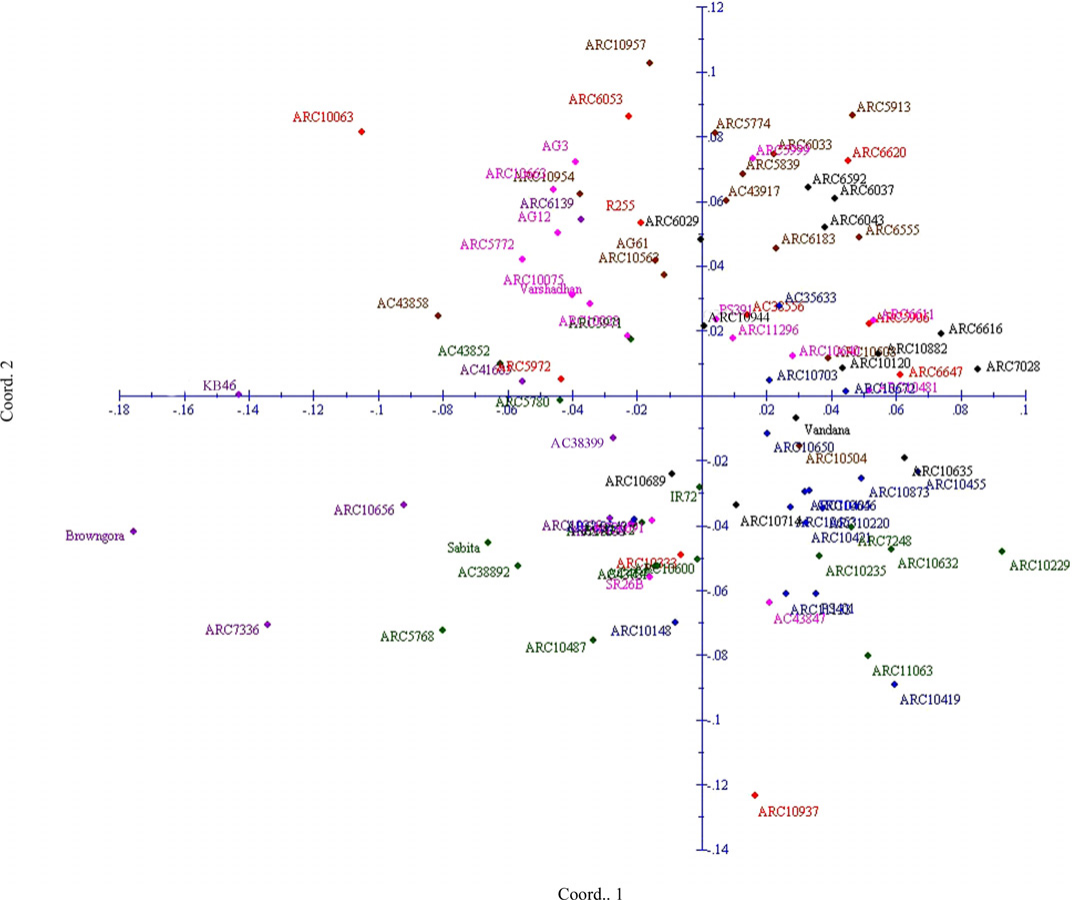
Principal Coordinate Analysis (PCoA) of rice genotypes based on trait linked SSRs. (genotypes represented in colors corresponding to the cluster observed in unrooted tree).

PCoA with SSR markers were used to determine the genetic relatedness among the genotypes (Fig 5). The first three axis of differentiation explained 19.98% of the total variation. In PCoA, genotypes were presented in colors corresponding to the clusters observed in unrooted tree. Similar to clusters analysis, PCoA also did not group the genotypes based on the location-specific and intermixing of colors across the coordinates were observed. However, improved genotypes were inclined to stay on the quadrant I and IV; tropical *japonica* AC 38892 and AC 38556 were on the opposite quadrant, indicating their dissimilarity.

#### Analysis of molecular variation (AMOVA) and Nei’s genetic distance

The assumed populations were subjected to AMOVA. AMOVA revealed that maximum variation was among individuals with greater variance (99%), while sub-populations were grouped together, the variance was only 1% and the F_st_ (0.016) was non-significant. The inbreeding co-efficient F_IS_ (-0.419) and F_IT_ (-0.396) were found to be 1.000. It indicated that individuals from different populations are genetically more closely related than within population. Nei’s genetic distance was estimated between assumed seven populations. The pairwise Nei’s genetic distance ranged from 0.0024 (lowest) between population of pop 6 and pop 7 to 0.0316 (highest) between population of pop 3 and pop 4.

#### Population structure

**Fig 6:**
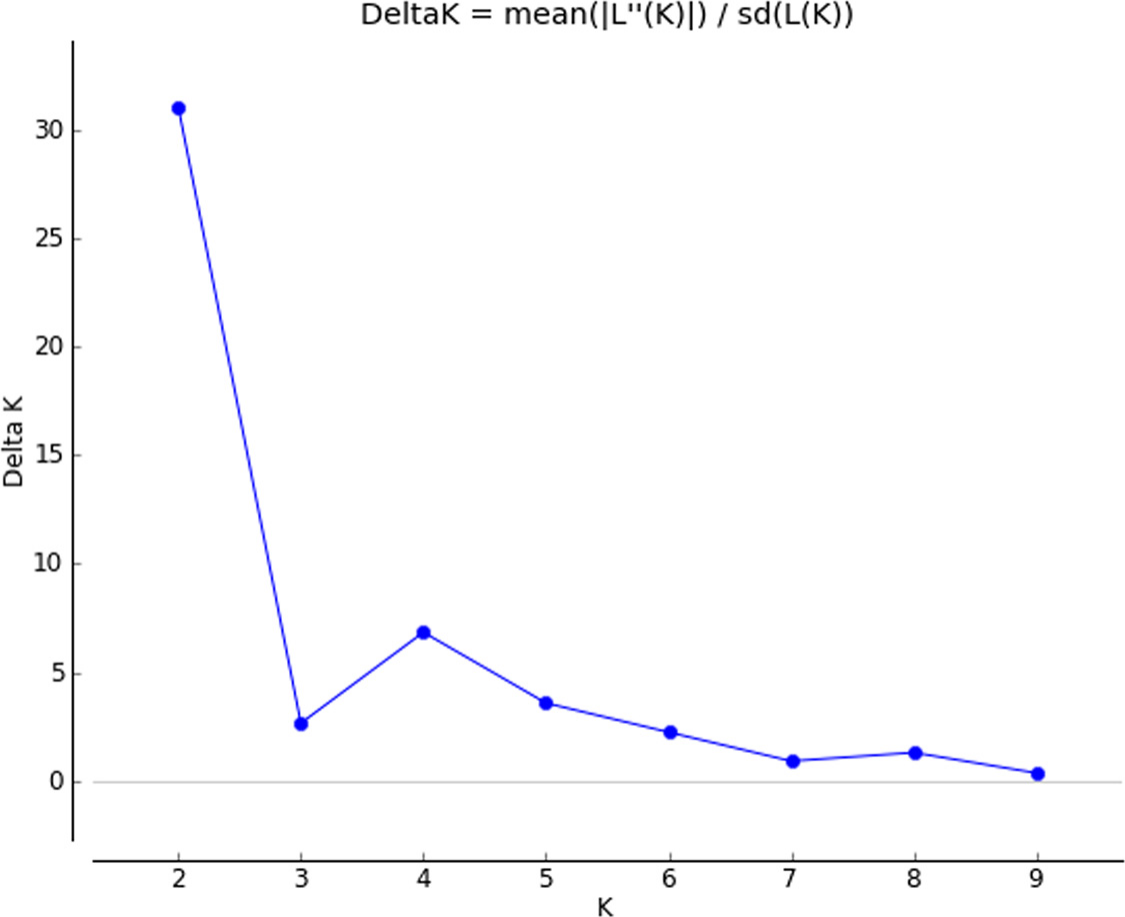
Estimation of population using LnP(D) derived delta K for determining optimum number of subpopulations.

**Fig 7:**
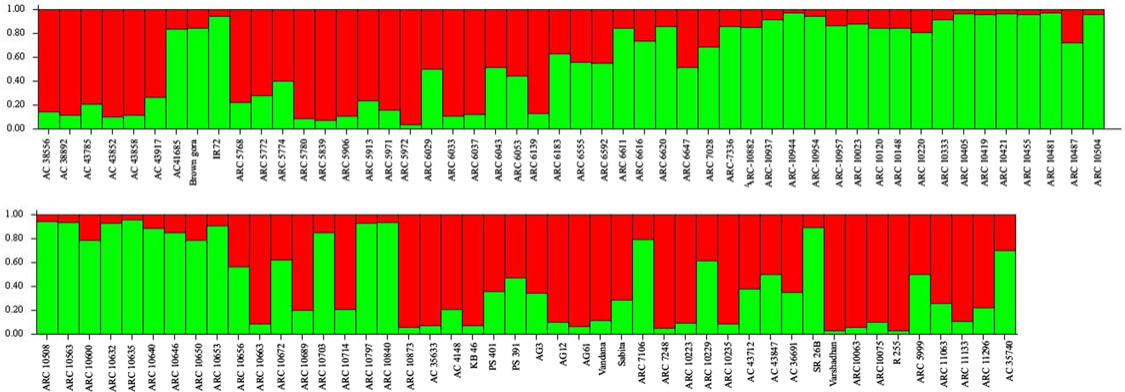
Distribution pattern of 96 rice genotypes based on SSRs within sub-populations.

STRUCTURE software was used to determine relationship among the genotypes studied and distinguished 96 genotypes into two populations with ΔK value of 31 (Fig 6). In population 1, 46 genotypes and rest of the 50 genotypes were grouped into population 2. Two tropical *japonica*, 11 improved lines, 26 ARC lines and seven landraces were grouped into one population with proportion of 47.91%. While, population 2 has the membership proportion of 52.08% consisted of 44 ARC lines, five landraces and one improved genotype IR72.By structure analysis, pure or admixture genotypes were categorized. The genotype with score >0.80 was considered as pure and <0.80 as admixture. Among the 96 genotypes, 27 genotypes were pure and 19 were admix in population 1, while 34 were pure and 16 were admix in population 2. In total, 61 were pure and 35 were identified as admixture and the same can be visualized in graphical representation (Fig 7). The fixation index (F_st_) values of two population ranged between 0.0218 (population 1) and 0.3397 (population 2), while allele frequency divergence between two population was 0.057.

#### Association of SSRs with early seedling vigor traits

The results of association analysis using GLM and MLM (Q+K) are presented in the Table 5. The structured association mapping revealed 16 and 10 marker-trait association for 12 and 11 ESV related parameters were observed by GLM and MLM approach respectively. GLM analysis of marker-trait association among the population revealed markers RM13 and RM334 with leaf length observed on 14 DAS located on chromosome 1 and 5 respectively. By accounting population structure and relative kinship effects (Q+K model), RM13 marker associated leaf length on 14 DAS with 7.3% of phenotypic variability. GLM and MLM analysis together they detected RM223 strongly associated with leaf length on 28 DAS with 10.3% and 8.4% phenotypic variability respectively. There were two common markers associated with leaf width on 14 DAS distributed over chromosome 8 and 7, of which RM230 on chromosome 8 had described 5.05% of the phenotypic variability. For root length on 14 DAS, two QTLs were detected among the population by both analyses, and RM3839 (chromosome 4) was associated with root length followed by RM230 (chromosome 8). RM249 and RM6091 on chromosome 4 and 8 had described 4.8% and 3.8% of the phenotypic variability respectively for root length on 28 DAS by GLM analysis. Followed by MLM, RM249 exhibited association with root length on 28 DAS with 5.04% of phenotypic variability. Three SSR markers (RM13, RM3348 and RM16) related to shoot length on 14 DAS were detected by GLM. Among them RM13 and RM16 accounting 6.05% and 5.14% phenotypic variability for shoot length detected by MLM. None of the markers were found to be associated with shoot length on 28 DAS and dry shoot weight on 14 DAS by GLM and MLM analyses. Marker RM341 was found to be associated with vigor index QTLs as detected by GLM and MLM with >4.5% phenotypic variability.

**Table 5:** Association of marker alleles with phenotypic traits of early seedling vigor in rice under direct seeded situation using GLM and MLM (Q+K) model.

Trait	Marker	Chromosome	GLM Model			MLM (Q+K) model		
			*F* ratio	p-value	R^2^	*F* ratio	p-value	R^2^
Leaf length (14)	RM13	1	6.65	0.01	0.07	6.82	0.01	0.07
	RM334	5	6.24	0.01	0.06	-	-	-
Leaf length (28)	RM223	8	11.58	0.00	0.10	8.85	0.00	0.08
	RM6	2	4.53	0.04	0.04	-	-	-
Leaf width (14)	RM230	8	5.20	0.02	0.05	4.97	0.03	0.05
	RM125	7	4.17	0.04	0.04	4.03	0.05	0.04
	RM218	3	3.91	0.05	0.04	-	-	-
No. of leaf (14)	RM224	11	5.90	0.02	0.06	-	-	-
	RM21	11	4.41	0.04	0.05	-	-	-
	RM13	5	4.11	0.05	0.04	-	-	-
Root length (14)	RM3839	4	4.60	0.03	0.04	5.10	0.03	0.05
	RM230	8	4.33	0.04	0.04	4.78	0.03	0.05
Root length (28)	RM249	5	5.35	0.02	0.05	4.95	0.03	0.05
	RM6091	11	4.19	0.04	0.04	-	-	-
Root weight (14)	RM7075	1	5.21	0.02	0.05	-	-	-
	RM249	5	4.82	0.03	0.05	4.74	0.03	0.05
Root weight (28)	RM250	2	4.06	0.05	0.04	3.92	0.05	0.04
Shoot length (14)	RM13	1	5.43	0.02	0.05	4.75	0.03	0.05
	RM334	5	5.26	0.02	0.05	-	-	-
	RM16	3	3.99	0.05	0.04	5.60	0.02	0.06
Shoot weight (28)	RM341	2	5.50	0.02	0.05	5.26	0.02	0.05
	RM224	11	4.10	0.05	0.04	4.23	0.04	0.04
Vigor index (14)	RM341	2	4.37	0.04	0.04	5.10	0.03	0.05
Vigor index (28)	RM341	2	4.35	0.04	0.05	4.85	0.03	0.05

## Discussion

Genetic variability is the key determinant for any breeding program. The sporadic reports on germplasm evaluation of landraces and genetics of ESV traits in rice encouraged us to take up this study. Traditional native landraces those grown under rainfed are competitive to weeds. By introduction of modern high yielding with low ESV semi dwarf varieties, weed competitive landraces are almost extinct and disappearing in farmers’ field. Therefore, the present experiment was executed to identify the suitable genotypes with high ESV and to find the loci responsible for those traits of the gene by association mapping. We began with a panel of 629 rice accessions (S1 Table). Landraces included in this study were from seven states of India *viz*., Assam, Odisha, Uttar Pradesh, Karnataka, Tamil Nadu, West Bengal and Bihar. The early seedling vigor trait distribution pattern of the panel of genotype studied on 14^th^, 28^th^ and 56^th^ days after sowing (DAS), exhibited that germination rate, number of leaves on 14 DAS, DW on 56 DAS, VI on 56 DAS and CGR distribution was asymmetrical. VI and DW on 56 DAS and CGR between 28 and 56 DAS exhibited maximum variability and portrayed that the population was heterogeneous.

### Spatial distribution of genotypes and variation of ESV traits

In this study, association between dry weight and shoot length or vigor index suggests that accumulation of dry weight is an important trait contributing for ESV. Cultivar-by-trait biplot analysis grouped the vectors of the variables of 14 and 28 DAS into a separate group (highly correlated). Similarly, variables of 56 DAS had formed another group (highly correlated). The reason might have been due to higher differences in the trait variability observed before 28 and 56 DAS. Further, it clarifies that the observation taken on 14 and 28 DAS was the reliable estimator to study ESV in rice than observed on 56 DAS. The position and perpendicular projection of genotypic points onto variable vectors grouped early vigor genotypes on 14 and 28 DAS in quadrant 2 and these genotypes were moderately vigorous on 56 DAS than genotypes captured in quadrant 1 and 4. Genotypes captured in quadrant 3, exhibited vigorous growth on 56 DAS with moderate early vigor than genotypes grouped in quadrant 1 and 4.

The PCA analysis of selected 96 genotypes under net house and field condition classified early vigor genotypes on right of the biplot and non-early vigorous genotypes on left side of the plot. This is evident from the genotypes like ‘Sabita’ (a known early vigor genotype) [32] positioned on right side with other early vigor genotypes *viz*., Varshadhan, Vandana, AC4387 and Pyari. In contrary, genotypes KB46, AC 38399 and ARC 10656 moved on the left side of the plot, where they exhibited reduced vigor index and dry weight.

### Diversity estimation, genetic relationships and grouping of genotypes

The genetic architecture of diverse rice accessions can be assessed by model based and distance based clustering approach using the SSRs or SNPs marker system [33–37]. This is the first such study where ESV trait linked SSR markers were utilized to assess genetic diversity and population structure in 96 germplasm lines of Indian rice collections. We are confident enough that, comprehensive analysis of trait linked SSR markers in such a diverse collection will be helpful for breeders to design breeding strategies for direct seeded situation with ESV.

The 39 SSRs generated a total of 128 alleles with an average of 3.28 alleles per locus (Table 4) and PIC value of 0.24. Comparison of our findings with previous studies in rice showed mixed results. Singh et al (2013) also observed a lower number of alleles (2.22) per locus and an average PIC value of 0.25 in 375 Indian rice collections. Similarly, Shah et al. [38] reported lower genetic diversity with an average of 2.75 allele per locus and an average PIC value of 0.38 from 40 Pakistan rice accessions. On the other hand, Behera et al. [39] also reported 4.69 alleles per locus with an average PIC value of 0.81 among the 36 landraces of having different therapeutic values from India. In the present context, reason for reduced number of alleles may be the use of trait linked SSR markers and the exclusion of monomorphic and spurious bands from analysis [35]. Several factors were reported to improve average PIC values of SSRs such as inclusion of diverse lines, size of collection, breeding behaviors of the species, genotypic method and location of markers in the genome [35]. Lower PIC value observed in the present study might be due to inclusion of large number of ARC accessions. One unique and nine rare alleles were observed in the present study. Unique allele can be considered for diagnostic of a particular genotype and increase in number of rare alleles contributes for overall genetic diversity. The proportion low or high frequency allele also contributes to overall genetic diversity. In our case of study, high frequency allele has 58.7% contribution over low frequency allele.

Genetic relatedness among the accessions was assessed on the basis of their clustering pattern in the unrooted tree. The unrooted tree grouped varieties into three major clusters with varying number of genotypes per cluster. The clustering pattern of genotypes obtained in the present study was found to be trait based grouping and no geographical isolation clustering has been observed. Inclusion of large number of ARC collections might be the reason for lack of geographical isolation. Accessions of subgroup 1b possessing 11 ESV traits measured on 14 and 28 DAS were grouped together with higher crop growth rate (CGR). The genotypes were grouped together under the subgroup1b were tall and possessed high vigor index (VI) as preferred for ESV. While, cluster 3 positioned opposite to subgroup 1b grouped the genotypes of least vigor type and exhibited inferiority for 11 ESV traits including vigor index. Singh et al. [35] also reported similar grouping pattern in Indian rice collections for biotic stress. The grouping pattern of the PCoA corresponded well with the pattern of Neighbor Joining Tree. Genotypes of subgroup 1b (deep blue color) tends to stay in quadrant 3, whereas, corresponding genotypes of cluster 3 (purple color) moved apart to the left extreme of PCoA plot. However, few exceptions were observed in position of genotypes compared to cluster analysis due to the complexity of the variations could not explained in two dimensional graphical representation, as the first two PCs explained the total variation less than 80% [40].

### Source of variation, population structure and marker-trait association

AMOVA study indicated higher partitioning of variation within individuals and lower portion existed among populations. Estimates of the fixation indices revealed that F_st_ indicates little divergence existing between subgroups of the population. F_IT_ is the overall inbreeding coefficient of an individual within the total population. Negative value of F_IT_ indicates higher proportion of heterozygote genotypes on the level of sub populations and all evaluated individuals as well. Negative F_IS_ value also suggests that there is an excess of heterozygotes in these loci (out breeding) compared with Hardy Weinberg Equilibrium (HWE) expectations and reflects the genetic variability of general population. The reason for heterozygotes might be inclusion of landraces in the present study and these genotypes might have heterozygous allele for the studied trait.

The model based approach by structure analysis revealed two subpopulations. The genetic structure of populations has been previously reported in rice to vary from 2 to 8 subpopulations [33, 37, 41–44]. Courtois et al. [33] and Das et al. [42] have detected two and four subgroups in their study population of 425 and 91 accessions of rice landraces respectively. Threshold level to identify accessions belonging to a specific subpopulation varies among different scientific groups of 60–80% [33, 45, 46]. In the present study, 35 landrace accessions were categorized as admixtures with the stringent threshold of 80%. Based on criterion of maximum membership probabilities, 96 accessions were grouped into two populations of 46 and 50 genotypes each. This finding is in accordance with the previous studies where they obtained two distinct subgroups in their core collection of rice [33, 47]. In the current rice collection panel, the use of trait linked SSR markers might also be the reason for the formation of less number of groups. Besides, grouping of the genotypes into two were noticed based on the traits of ESV observed on 14^th^and 28^th^ DAS. Between the two sub-populations, second sub-population exhibited higher shoot length, root length, number of leaves, leaf length, dry weight and VI on both DAS than the sub-population-1. However, genotypes of admixtures were observed to have superior in early vigor trait than pure lines. Since, this study includes large set of landraces, which might have diverse ancestry still possessing heterozygosity for the trait studied in the present experiment.

**Fig 8:**
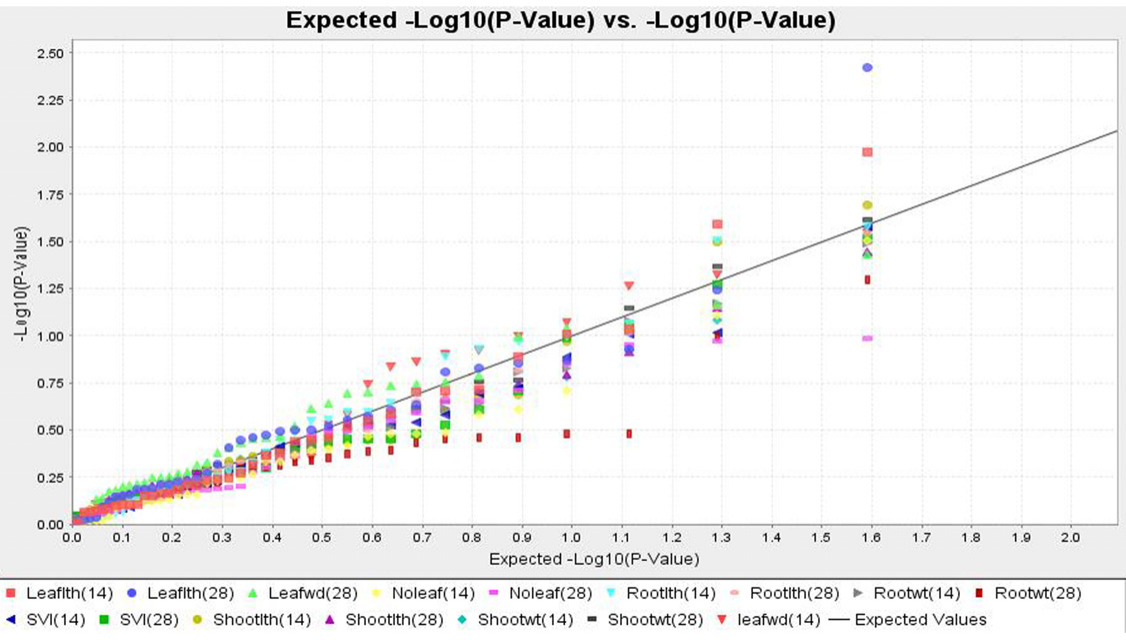
Quantile-Quantile (QQ) plot and distribution of SSR-trait association.

The application of association mapping for ESV will be helpful in deciding the molecular markers to be used in stacking the multiple QTLs responsible for this trait. The significant SSR markers of GLM explained on an average of 5.0% and MLM demonstrated 5.2% of phenotypic variation. Maximum 10.3% and 8.4% phenotypic variation was explained by RM223 for leaf length on 28 DAS by GLM and MLM respectively. Similarly, Q-Q plot exhibits the significant association markers for leaf length on 28 DAS (Fig 8). A QTL for leaf length on 14 DAS detected in the present study on chromosome 1 (RM13) was observed in both GLM and MLM models.

SSR genomic region RM230 and RM125 mapped on chromosome 8 and 7 were significantly associated with QTL controlling leaf width variation on 14 DAS. Increasing leaf width by utilizing favorable allele at this locus would likely to be useful for increasing photosynthetic area of seedling and weed smothering ability. On the other hand, the same marker (RM230) allele was significantly associated with root length on 14 DAS. Previously, Zhang et al. [19] reported this marker on chromosome 8 as contributing to root length andRM3839 was also observed to be significantly associated with root length on 14 DAS by both GLM and MLM approach. QTLs *qRL-1* and *qRL-2* of root length were reported on chromosome 1 and 2 respectively from RIL population of Xiushui 79/C-Bao [48]. The root dry weight QTL was detected on chromosome 5 (by linked marker allele RM249) on 14 DAS coincided with QTL of root length on 28 DAS. For root dry weight on 28 DAS, significant association of RM250 was found by both GLM and MLM on chromosome 2. Further, a QTL *qRDW10-1*was identified for root dry weight on chromosome of 10 with LOD score of 5.6 from RIL population of Zhenshan 97/Ming hui63 [21].

Two significant QTLs (chromosome 1 and 3) were identified for shoot length on 14 DAS. Marker alleles RM13 on chromosome 1 and RM16 on chromosome 3 were positively associated with shoot length on 14 DAS. Moreover, previous report of Zhang et al. [19] supports the association of genomic region RM13 with QTLs for shoot length. Hence, selection of these alleles can be expected to result in genetic improvement for this trait to obtain vigorous growth in the early stage of seedling under direct seeded condition. Cairns et al. [28] observed a QTL *qSL-2*for shoot length on chromosome 2 from back cross population of Vandana/Moroberekan. In our study, allele RM224 located on chromosome 11 was significantly associated with shoot dry weight on 28 DAS and another tightly linked marker allele (RM341) showed a stronger association with the same trait and detected by both GLM and MLM on chromosome 2. However, Zhou et al. [27] and Cui et al. [21] observed QTL *qFV-5-1* and *qDW5* for shoot dry weight on chromosome 5 from Lemont/Teqing and IR64/Azucena mapping population respectively. Interestingly, RM341 coincided with QTLs for vigor index on chromosome 2 at 14 and 28 DAS and showed a stronger association detected by both GLM and MLM. Conversely Diwan et al. [23] observed QTL *qVI* on chromosome 5 with LOD score of 4.4. Therefore, marker-assisted selection in favor of RM341 could increase the shoot length and vigor index and might also contribute favorably to grain yield and biomass under direct seeded conditions.

## Conclusion

Screening large number of accessions in the present study for early seedling vigor brings out valuable information. We conclude from this study that the vigor index should be studied on 14^th^ and 28^th^ DAS. The genotypes utilized for studying diversity pattern by employing trait-linked markers have categorized early vigor and non-early vigor genotypes. The results of distance based approach and principal coordinate analysis were in accordance with model based structure analysis. Further, the study has identified significantly associated markers and markers with pleiotropic effects indicating that association mapping served as an effective tool. The putative QTLs, identified in this study, need to be validated before they could be applied for marker assisted breeding in rice. Genotypes identified from this study possessing favorable alleles can be utilized for improving early seedling vigor after functionally characterizing them.

## Supporting Information

S1 Fig (a-h). Distribution and differences for shoot length, number of leaves, root length, DW, VI, G. rate, AGR, CGR and RGR under direct seeded situation during 14, 28 and 56 DAS among 629 rice genotypes. Acronyms used: DW-shoot dry weight, VI-vigor index, G. rate-Germination rate, AGR-Absolute growth rate, CGR-Crop growth rate, RGR-Relative growth rate and DAS-days after sowing.
(TIF)

S1 Table The list of the rice genotypes used in the study. (DOCX)
