# Supplementary figures and images for: Population Structure, Diversity and Trait Association Analysis in Rice (Oryza sativa L.) Germplasm for Early Seedling Vigor (ESV) Using Trait Linked SSR Markers
Source: PLoS One. 2016 Mar 31;11(3):e0152406. doi: 10.1371/journal.pone.0152406 (PMC4816567; doi:10.1371/journal.pone.0152406)

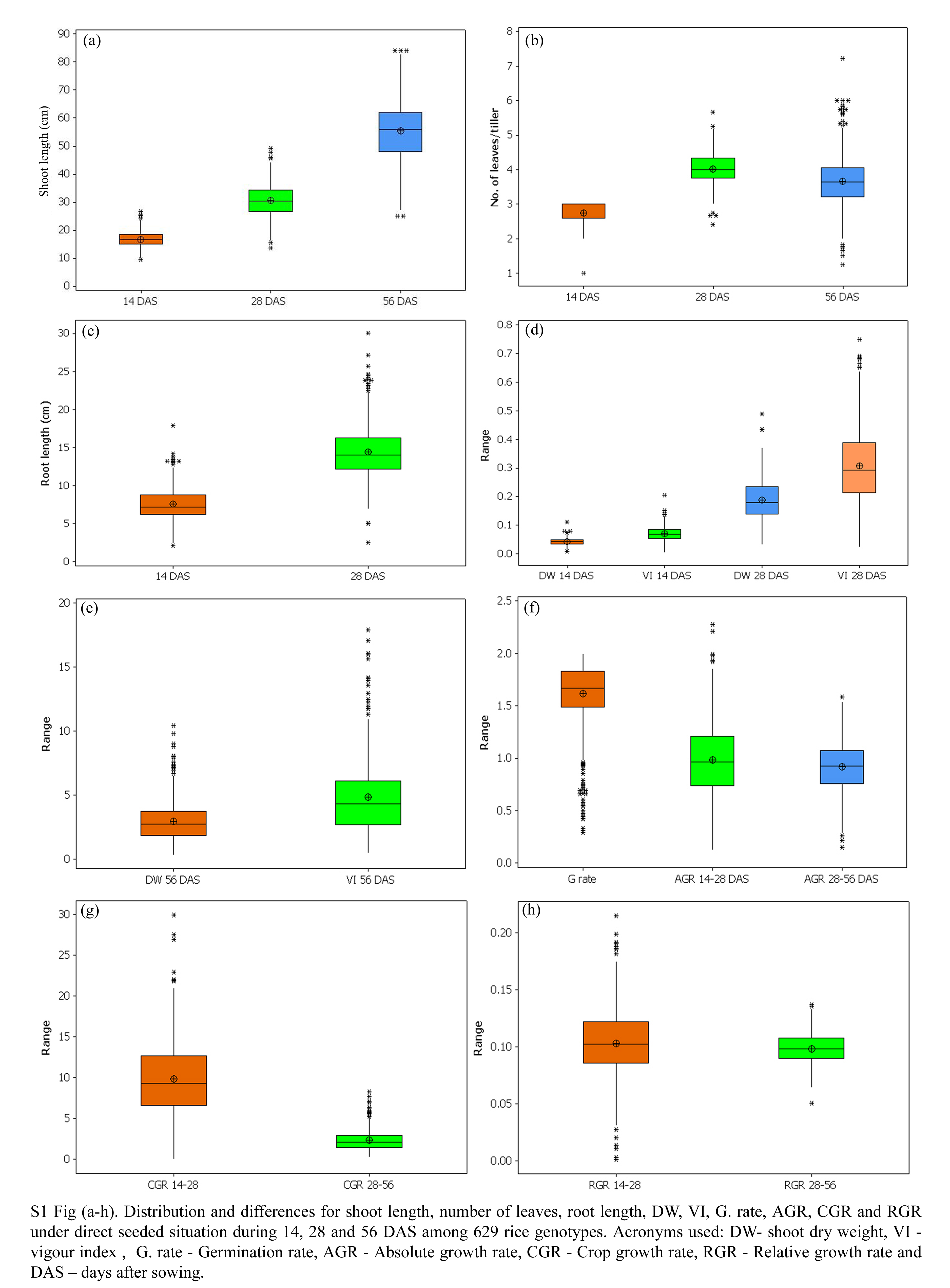

Supplement: S1 Fig — (a-h). Distribution and differences for shoot length, number of leaves, root length, DW, VI, G. rate, AGR, CGR and RGR under direct seeded situation during 14, 28 and 56 DAS among 629 rice genotypes. Acronyms used: DW-shoot dry weight, VI-vigor index, G. rate-Germination rate, AGR-Absolute growth rate, CGR-Crop growth rate, RGR-Relative growth rate and DAS-days after sowing. (TIF) [file pone.0152406.s001.tif]
