# Supplementary material for: Population Structure, Diversity and Trait Association Analysis in Rice (Oryza sativa L.) Germplasm for Early Seedling Vigor (ESV) Using Trait Linked SSR Markers
Source: PLoS One. 2016 Mar 31;11(3):e0152406. doi: 10.1371/journal.pone.0152406 (PMC4816567; doi:10.1371/journal.pone.0152406)
Supplement: S1 Table — (DOCX) [file pone.0152406.s002.docx]

**S1 Table. The list of the rice genotypes used in the study**

| **S.No** | **Accession/**  **Variety** | **Land races/ Variety** | **State** | **Country** | **S.No** | **Accession/**  **Variety** | **Land races/ variety** | **State** | **Country** |
| --- | --- | --- | --- | --- | --- | --- | --- | --- | --- |
| 1 | AC 36308 | Land race | Assam | India | 316 | ARC 10960 | Land race | Assam | India |
| 2 | AC 36763 | Land race | WB | India | 317 | ARC 10023^🞺^ | Land race | Assam | India |
| 3 | AC 38392 | *Tropical Japonica* | IRRI | Philippines | 318 | ARC 10059 | Land race | Assam | India |
| 4 | AC 38407 | *Tropical Japonica* | IRRI | Philippines | 319 | ARC 10061 | Land race | Assam | India |
| 5 | AC 38422 | *Tropical Japonica* | IRRI | Philippines | 320 | ARC 10062 | Land race | Assam | India |
| 6 | AC 38448 | *Tropical Japonica* | IRRI | Philippines | 321 | ARC 10088 | Land race | Assam | India |
| 7 | AC 38465 | *Tropical Japonica* | IRRI | Philippines | 322 | ARC 10090 | Land race | Assam | India |
| 8 | AC 38474 | *Tropical Japonica* | IRRI | Philippines | 323 | ARC 10118 | Land race | Assam | India |
| 9 | AC 38517 | *Tropical Japonica* | IRRI | Philippines | 324 | ARC 10120^🞺^ | Land race | Assam | India |
| 10 | AC 38556^🞺^ | *Tropical Japonica* | IRRI | Philippines | 325 | ARC 10148^🞺^ | Land race | Assam | India |
| 11 | AC 38570 | *Tropical Japonica* | IRRI | Philippines | 326 | ARC 10152 | Land race | Assam | India |
| 12 | AC 38571 | *Tropical Japonica* | IRRI | Philippines | 327 | ARC 10157 | Land race | Assam | India |
| 13 | AC 38659 | *Tropical Japonica* | IRRI | Philippines | 328 | ARC 10168 | Land race | Assam | India |
| 14 | AC 38684 | *Tropical Japonica* | IRRI | Philippines | 329 | ARC 10187 | Land race | Assam | India |
| 15 | AC 38732 | *Tropical Japonica* | IRRI | Philippines | 330 | ARC 10194 | Land race | Assam | India |
| 16 | AC 38758 | *Tropical Japonica* | IRRI | Philippines | 331 | ARC 10197 | Land race | Assam | India |
| 17 | AC 38892 | *Tropical Japonica* | IRRI | Philippines | 332 | ARC 10162 | Land race | Assam | India |
| 18 | AC 42374 | Improved variety | South America | Suriname | 333 | ARC 10156 | Land race | Assam | India |
| 19 | AC 42375 | Improved variety | South America | Suriname | 334 | ARC 10171 | Land race | Assam | India |
| 20 | AC 42376 | Improved variety | South America | Suriname | 335 | ARC 10191 | Land race | Assam | India |
| 21 | AC 42379 | Improved variety | South America | Suriname | 336 | ARC 10178 | Land race | Assam | India |
| 22 | AC 42380 | Improved variety | South America | Suriname | 337 | ARC 10220^🞺^ | Land race | Assam | India |
| 23 | AC 42381 | Improved variety | South America | Suriname | 338 | ARC 10222 | Land race | Assam | India |
| 24 | AC 43785^🞺^ | Land race | Odisha | India | 339 | ARC 10223^🞺^ | Land race | Assam | India |
| 25 | AC 43801 | Land race | Odisha | India | 340 | ARC 10229^🞺^ | Land race | Assam | India |
| 26 | AC 43852^🞺^ | Land race | Tamil Nadu | India | 341 | ARC 10235^🞺^ | Land race | Assam | India |
| 27 | AC 43858^🞺^ | Land race | Tamil Nadu | India | 342 | ARC 10243 | Land race | Assam | India |
| 28 | AC 43872 | Improved variety | IRRI | Philippines | 343 | ARC 10244 | Land race | Assam | India |
| 29 | AC 43876 | Improved variety | IRRI | Philippines | 344 | ARC 10245 | Land race | Assam | India |
| 30 | AC 43884 | Improved variety | British guiana | Africa | 345 | ARC 10248 | Land race | Assam | India |
| 31 | AC 43891 | Land race | Karnataka | India | 346 | ARC 10258 | Land race | Assam | India |
| 32 | AC 43901 | Land race | Odisha | India | 347 | ARC 10259 | Land race | Assam | India |
| 33 | AC 43903 | Land race | Odisha | India | 348 | ARC 10260 | Land race | Assam | India |
| 34 | AC 43909 | Land race | Karnataka | India | 349 | ARC 10262 | Land race | Assam | India |
| 35 | AC 43917^🞺^ | Land race | Karnataka | India | 350 | ARC 10264 | Land race | Assam | India |
| 36 | AC 44091 | Improved variety | Karnataka | India | 351 | ARC 10266 | Land race | Assam | India |
| 37 | AC40452 | Land race | Uttar Pradesh | India | 352 | ARC 10269 | Land race | Assam | India |
| 38 | AC41685^🞺^ | Land race | Uttar Pradesh | India | 353 | ARC 10270 | Land race | Assam | India |
| 39 | Browngora^🞺^ | Improved variety | Bihar | India | 354 | ARC 10271 | Land race | Assam | India |
| 40 | AC 41299 | Land race | - | India | 355 | ARC 10281 | Land race | Assam | India |
| 41 | AC 43633 | Land race | West Bengal | India | 356 | ARC 10276 | Land race | Assam | India |
| 42 | Dular | Improved variety | - | India | 357 | ARC 10287 | Land race | Assam | India |
| 43 | IR72^🞺^ | Improved variety | IRRI | Philippines | 358 | ARC 10304 | Land race | Assam | India |
| 44 | N22 | Improved variety | UP/Bihar | India | 359 | ARC 10314 | Land race | Assam | India |
| 45 | Sabita^🞺^ | Improved variety | West Bengal | India | 360 | ARC 10317 | Land race | Assam | India |
| 46 | Solumpiket | Improved variety | - | India | 361 | ARC 10321 | Land race | Assam | India |
| 47 | Vandana^🞺^ | Improved variety | - | India | 362 | ARC 10333^🞺^ | Land race | Assam | India |
| 48 | ARC 5751 | Land race | Assam | India | 363 | ARC 10342 | Land race | Assam | India |
| 49 | ARC 5757 | Land race | Assam | India | 364 | ARC 10344 | Land race | Assam | India |
| 50 | ARC 5758 | Land race | Assam | India | 365 | ARC 10363 | Land race | Assam | India |
| 51 | ARC 5759 | Land race | Assam | India | 366 | ARC 10379 | Land race | Assam | India |
| 52 | ARC 5764 | Land race | Assam | India | 367 | ARC 10392 | Land race | Assam | India |
| 53 | ARC 5767 | Land race | Assam | India | 368 | ARC 10393 | Land race | Assam | India |
| 54 | ARC 5768^🞺^ | Land race | Assam | India | 369 | ARC 10399 | Land race | Assam | India |
| 55 | ARC 5769 | Land race | Assam | India | 370 | ARC 10405^🞺^ | Land race | Assam | India |
| 56 | ARC 5772^🞺^ | Land race | Assam | India | 371 | ARC 10416 | Land race | Assam | India |
| 57 | ARC 5774^🞺^ | Land race | Assam | India | 372 | ARC 10419^🞺^ | Land race | Assam | India |
| 58 | ARC 5776 | Land race | Assam | India | 373 | ARC 10424 | Land race | Assam | India |
| 59 | ARC 5778 | Land race | Assam | India | 374 | ARC 10426 | Land race | Assam | India |
| 60 | ARC 5779 | Land race | Assam | India | 375 | ARC 10438 | Land race | Assam | India |
| 61 | ARC 5780^🞺^ | Land race | Assam | India | 376 | ARC 10444 | Land race | Assam | India |
| 62 | ARC 5781 | Land race | Assam | India | 377 | ARC 10468 | Land race | Assam | India |
| 63 | ARC 5783 | Land race | Assam | India | 378 | ARC 10491 | Land race | Assam | India |
| 64 | ARC 5784 | Land race | Assam | India | 379 | ARC 10421^🞺^ | Land race | Assam | India |
| 65 | ARC 5786 | Land race | Assam | India | 380 | ARC 10446 | Land race | Assam | India |
| 66 | ARC 5787 | Land race | Assam | India | 381 | ARC 10450 | Land race | Assam | India |
| 67 | ARC 5791 | Land race | Assam | India | 382 | ARC 10451 | Land race | Assam | India |
| 68 | ARC 5793 | Land race | Assam | India | 383 | ARC 10455^🞺^ | Land race | Assam | India |
| 69 | ARC 5795 | Land race | Assam | India | 384 | ARC 10457 | Land race | Assam | India |
| 70 | ARC 5797 | Land race | Assam | India | 385 | ARC 10459 | Land race | Assam | India |
| 71 | ARC 5799 | Land race | Assam | India | 386 | ARC 10471 | Land race | Assam | India |
| 72 | ARC 5801 | Land race | Assam | India | 387 | ARC 10481^🞺^ | Land race | Assam | India |
| 73 | ARC 5813 | Land race | Assam | India | 388 | ARC 10487^🞺^ | Land race | Assam | India |
| 74 | ARC 5823 | Land race | Assam | India | 389 | ARC 10493 | Land race | Assam | India |
| 75 | ARC 5828 | Land race | Assam | India | 390 | ARC 10504^🞺^ | Land race | Assam | India |
| 76 | ARC 5832 | Land race | Assam | India | 391 | ARC 10505 | Land race | Assam | India |
| 77 | ARC 5833 | Land race | Assam | India | 392 | ARC 10508^🞺^ | Land race | Assam | India |
| 78 | ARC 5839^🞺^ | Land race | Assam | India | 393 | ARC 10518 | Land race | Assam | India |
| 79 | ARC 5840 | Land race | Assam | India | 394 | ARC 10519 | Land race | Assam | India |
| 80 | ARC 5841 | Land race | Assam | India | 395 | ARC 10525 | Land race | Assam | India |
| 81 | ARC 5842 | Land race | Assam | India | 396 | ARC 10527 | Land race | Assam | India |
| 82 | ARC 7135 | Land race | Assam | India | 397 | ARC 10544 | Land race | Assam | India |
| 83 | ARC 5846 | Land race | Assam | India | 398 | ARC 10563^🞺^ | Land race | Assam | India |
| 84 | ARC 5848 | Land race | Assam | India | 399 | ARC 10571 | Land race | Assam | India |
| 85 | ARC 5850 | Land race | Assam | India | 400 | ARC 10595 | Land race | Assam | India |
| 86 | ARC 5906^🞺^ | Land race | Assam | India | 401 | ARC 10599 | Land race | Assam | India |
| 87 | ARC 5911 | Land race | Assam | India | 402 | ARC 10600^🞺^ | Land race | Assam | India |
| 88 | ARC 5912 | Land race | Assam | India | 403 | ARC 10601 | Land race | Assam | India |
| 89 | ARC 5913^🞺^ | Land race | Assam | India | 404 | ARC 10603 | Land race | Assam | India |
| 90 | ARC 5914 | Land race | Assam | India | 405 | ARC 10604 | Land race | Assam | India |
| 91 | ARC 5918 | Land race | Assam | India | 406 | ARC 10606 | Land race | Assam | India |
| 92 | ARC 5920 | Land race | Assam | India | 407 | ARC 10608 | Land race | Assam | India |
| 93 | ARC 5922 | Land race | Assam | India | 408 | ARC 10609 | Land race | Assam | India |
| 94 | ARC 5927 | Land race | Assam | India | 409 | ARC 10610 | Land race | Assam | India |
| 95 | ARC 5928 | Land race | Assam | India | 410 | ARC 10611 | Land race | Assam | India |
| 96 | ARC 5937 | Land race | Assam | India | 411 | ARC 10612 | Land race | Assam | India |
| 97 | ARC 5940 | Land race | Assam | India | 412 | ARC 10614 | Land race | Assam | India |
| 98 | ARC 5944 | Land race | Assam | India | 413 | ARC 10616 | Land race | Assam | India |
| 99 | ARC 5946 | Land race | Assam | India | 414 | ARC 10617 | Land race | Assam | India |
| 100 | ARC 5951 | Land race | Assam | India | 415 | ARC 10618 | Land race | Assam | India |
| 101 | ARC 5956 | Land race | Assam | India | 416 | ARC 10619 | Land race | Assam | India |
| 102 | ARC 5965 | Land race | Assam | India | 417 | ARC 10625 | Land race | Assam | India |
| 103 | ARC 5971^🞺^ | Land race | Assam | India | 418 | ARC 10632^🞺^ | Land race | Assam | India |
| 104 | ARC 5972^🞺^ | Land race | Assam | India | 419 | ARC 10635^🞺^ | Land race | Assam | India |
| 105 | ARC 5973 | Land race | Assam | India | 420 | ARC 10636 | Land race | Assam | India |
| 106 | ARC 5975 | Land race | Assam | India | 421 | ARC 10640^🞺^ | Land race | Assam | India |
| 107 | ARC 5976 | Land race | Assam | India | 422 | ARC 10645 | Land race | Assam | India |
| 108 | ARC 5977 | Land race | Assam | India | 423 | ARC 10646^🞺^ | Land race | Assam | India |
| 109 | ARC 5982 | Land race | Assam | India | 424 | ARC 10647 | Land race | Assam | India |
| 110 | ARC 5985 | Land race | Assam | India | 425 | ARC 10650^🞺^ | Land race | Assam | India |
| 111 | ARC 5993 | Land race | Assam | India | 426 | ARC 10651 | Land race | Assam | India |
| 112 | ARC 5994 | Land race | Assam | India | 427 | ARC 10653^🞺^ | Land race | Assam | India |
| 113 | ARC 5995 | Land race | Assam | India | 428 | ARC 10654 | Land race | Assam | India |
| 114 | ARC 5999^🞺^ | Land race | Assam | India | 429 | ARC 10655 | Land race | Assam | India |
| 115 | ARC 6000 | Land race | Assam | India | 430 | ARC 10656^🞺^ | Land race | Assam | India |
| 116 | ARC 6001 | Land race | Assam | India | 431 | ARC 10657 | Land race | Assam | India |
| 117 | ARC 6004 | Land race | Assam | India | 432 | ARC 10661 | Land race | Assam | India |
| 118 | ARC 6005 | Land race | Assam | India | 433 | ARC 10663^🞺^ | Land race | Assam | India |
| 119 | ARC 6006 | Land race | Assam | India | 434 | ARC 10664 | Land race | Assam | India |
| 120 | ARC 6007 | Land race | Assam | India | 435 | ARC 10666 | Land race | Assam | India |
| 121 | ARC 6009 | Land race | Assam | India | 436 | ARC 10667 | Land race | Assam | India |
| 122 | ARC 6017 | Land race | Assam | India | 437 | ARC 10669 | Land race | Assam | India |
| 123 | ARC 6018 | Land race | Assam | India | 438 | ARC 10670 | Land race | Assam | India |
| 124 | ARC 6023 | Land race | Assam | India | 439 | ARC 10672^🞺^ | Land race | Assam | India |
| 125 | ARC 6025 | Land race | Assam | India | 440 | ARC 10682 | Land race | Assam | India |
| 126 | ARC 6026 | Land race | Assam | India | 441 | ARC 10689^🞺^ | Land race | Assam | India |
| 127 | ARC 6027 | Land race | Assam | India | 442 | ARC 10690 | Land race | Assam | India |
| 128 | ARC 6029^🞺^ | Land race | Assam | India | 443 | ARC 10695 | Land race | Assam | India |
| 129 | ARC 6033^🞺^ | Land race | Assam | India | 444 | ARC 10696 | Land race | Assam | India |
| 130 | ARC 6035 | Land race | Assam | India | 445 | ARC 10698 | Land race | Assam | India |
| 131 | ARC 6037^🞺^ | Land race | Assam | India | 446 | ARC 10699 | Land race | Assam | India |
| 132 | ARC 6038 | Land race | Assam | India | 447 | ARC 10700 | Land race | Assam | India |
| 133 | ARC 6039 | Land race | Assam | India | 448 | ARC 10702 | Land race | Assam | India |
| 134 | ARC 6040 | Land race | Assam | India | 449 | ARC 10703^🞺^ | Land race | Assam | India |
| 135 | ARC 6043^🞺^ | Land race | Assam | India | 450 | ARC 10714^🞺^ | Land race | Assam | India |
| 136 | ARC 6053^🞺^ | Land race | Assam | India | 451 | ARC 10744 | Land race | Assam | India |
| 137 | ARC 6058 | Land race | Assam | India | 452 | ARC 10753 | Land race | Assam | India |
| 138 | ARC 6060 | Land race | Assam | India | 453 | ARC 10776 | Land race | Assam | India |
| 139 | ARC 6076 | Land race | Assam | India | 454 | ARC 10790 | Land race | Assam | India |
| 140 | ARC 6082 | Land race | Assam | India | 455 | ARC 10792 | Land race | Assam | India |
| 141 | ARC 6088 | Land race | Assam | India | 456 | ARC 10797 | Land race | Assam | India |
| 142 | ARC 6091 | Land race | Assam | India | 457 | ARC 10827 | Land race | Assam | India |
| 143 | ARC 6093 | Land race | Assam | India | 458 | ARC 10834 | Land race | Assam | India |
| 144 | ARC 6096 | Land race | Assam | India | 459 | ARC 10838 | Land race | Assam | India |
| 145 | ARC 6097 | Land race | Assam | India | 460 | ARC 10840^🞺^ | Land race | Assam | India |
| 146 | ARC 6099 | Land race | Assam | India | 461 | ARC 10841 | Land race | Assam | India |
| 147 | ARC 6101 | Land race | Assam | India | 462 | ARC 10844 | Land race | Assam | India |
| 148 | ARC 6102 | Land race | Assam | India | 463 | ARC 10843 | Land race | Assam | India |
| 149 | ARC 6110 | Land race | Assam | India | 464 | ARC 10845 | Land race | Assam | India |
| 150 | ARC 6115 | Land race | Assam | India | 465 | ARC 10846 | Land race | Assam | India |
| 151 | ARC 6117 | Land race | Assam | India | 466 | ARC 10847 | Land race | Assam | India |
| 152 | ARC 6123 | Land race | Assam | India | 467 | ARC 10851 | Land race | Assam | India |
| 153 | ARC 6127 | Land race | Assam | India | 468 | ARC 10857 | Land race | Assam | India |
| 154 | ARC 6130 | Land race | Assam | India | 469 | ARC 10873 | Land race | Assam | India |
| 155 | ARC 6135 | Land race | Assam | India | 470 | ARC 10878 | Land race | Assam | India |
| 156 | ARC 6139^🞺^ | Land race | Assam | India | 471 | AC 35004 | Land race | ORS | India |
| 157 | ARC 6143 | Land race | Assam | India | 472 | AC 35633^🞺^ | Land race | Odisha | India |
| 158 | ARC 6144 | Land race | Assam | India | 473 | AC 37938 | Land race | W.B. | India |
| 159 | ARC 6147 | Land race | Assam | India | 474 | AC 4148^🞺^ | Improved variety | - | USA |
| 160 | ARC 6153 | Land race | Assam | India | 475 | AC 41620 | Land race | Bihar | India |
| 161 | ARC 6154 | Land race | Assam | India | 476 | AC 43967 | Land race | - | India |
| 162 | ARC 6156 | Land race | Assam | India | 477 | AC 44012 | Land race | - | India |
| 163 | ARC 6161 | Land race | Assam | India | 478 | AC 44018 | Land race | - | India |
| 164 | ARC 6170 | Land race | Assam | India | 479 | AC 44052 | Land race | - | India |
| 165 | ARC 6171 | Land race | Assam | India | 480 | AC 44057 | Land race | - | India |
| 166 | ARC 6172 | Land race | Assam | India | 481 | AC 44071 | Land race | - | India |
| 167 | ARC 6173 | Land race | Assam | India | 482 | AC 44087 | Land race | Odisha | India |
| 168 | ARC 6174 | Land race | Assam | India | 483 | AC 44099 | Land race | - | India |
| 169 | ARC 6175 | Land race | Assam | India | 484 | AC 44100 | Land race | - | India |
| 170 | ARC 6180 | Land race | Assam | India | 485 | ANJALI | Improved variety | NRRI, Odisha | India |
| 171 | ARC 6183^🞺^ | Land race | Assam | India | 486 | F5-444-2-2-5 | Improved variety | Odisha | India |
| 172 | ARC 6202 | Land race | Assam | India | 487 | F5-444-3-1-1 | Improved variety | Odisha | India |
| 173 | ARC 6206 | Land race | Assam | India | 488 | F5-444-3-1-1-2 | Improved variety | Odisha | India |
| 174 | ARC 6218 | Land race | Assam | India | 489 | F5-444-3-1-1-3 | Improved variety | Odisha | India |
| 175 | ARC 6220 | Land race | Assam | India | 490 | Piyari | Improved variety | Odisha | India |
| 176 | ARC 6230 | Land race | Assam | India | 491 | CR Dhan 201 | Improved variety | Odisha | India |
| 177 | ARC 6234 | Land race | Assam | India | 492 | CR Dhan 204 | Improved variety | Odisha | India |
| 178 | ARC 6235 | Land race | Assam | India | 493 | CR Dhan 202 | Improved variety | Odisha | India |
| 179 | ARC 6237 | Land race | Assam | India | 494 | CR Dhan 601 | Improved variety | Odisha | India |
| 180 | ARC 6249 | Land race | Assam | India | 495 | Sharvesh | Improved variety | Tamil Nadu | India |
| 181 | ARC 6555^🞺^ | Land race | Assam | India | 496 | CO 51 | Improved variety | Tamil Nadu | India |
| 182 | ARC 6557 | Land race | Assam | India | 497 | AC 11322 | Land race | Assam | India |
| 183 | ARC 6558 | Land race | Assam | India | 498 | AC 34245 | Land race | Odisha | India |
| 184 | ARC 6562 | Land race | Assam | India | 499 | BKN 6806-56 | Improved variety | - | - |
| 185 | ARC 6567 | Land race | Assam | India | 500 | BRRI 337 | Improved variety | - | Bangladesh |
| 186 | ARC 6571 | Land race | Assam | India | 501 | BRRI 280 | Improved variety | - | Bangladesh |
| 187 | ARC 6581 | Land race | Assam | India | 502 | BRRI 673 | Improved variety | - | Bangladesh |
| 188 | ARC 6582 | Land race | Assam | India | 503 | POKKALI | Land race | Kerala | India |
| 189 | ARC 6588 | Land race | Assam | India | 504 | AG 1-46 | Improved variety | - | - |
| 190 | ARC 6591 | Land race | Assam | India | 505 | Bhirpala | - | - | - |
| 191 | ARC 6592^🞺^ | Land race | Assam | India | 506 | Ching Tai Chan | - | - | - |
| 192 | ARC 6595 | Land race | Assam | India | 507 | Kalarata 2-18 | Land race | - | India |
| 193 | ARC 6598 | Land race | Assam | India | 508 | Som | - | - | - |
| 194 | ARC 6603 | Land race | Assam | India | 509 | Pusa Basmati 1 | Improved variety | IARI | India |
| 195 | ARC 6605 | Land race | Assam | India | 510 | Pokkali | Land race | Kerala | India |
| 196 | ARC 6606 | Land race | Assam | India | 511 | Azucena | Improved variety | IRRI | Philippines |
| 197 | ARC 6608 | Land race | Assam | India | 512 | Pokkali | Land race | Kerala | India |
| 198 | ARC 6609 | Land race | Assam | India | 513 | PCR 92093-56-2-B | Improved variety | - | India |
| 199 | ARC 6611^🞺^ | Land race | Assam | India | 514 | AC 43949 | - | - | India |
| 200 | ARC 6612 | Land race | Assam | India | 515 | AC 43966 | - | - | India |
| 201 | ARC 6616^🞺^ | Land race | Assam | India | 516 | AC 44048 | - | - | India |
| 202 | ARC 6617 | Land race | Assam | India | 517 | AC 44049 | - | - | India |
| 203 | ARC 6620^🞺^ | Land race | Assam | India | 518 | AC 44050 | - | - | India |
| 204 | ARC 6622 | Land race | Assam | India | 519 | AC 44055 | - | - | India |
| 205 | ARC 6623 | Land race | Assam | India | 520 | AC 44086 | - | - | India |
| 206 | ARC 6625 | Land race | Assam | India | 521 | AC 44098 | - | - | India |
| 207 | ARC 6628 | Land race | Assam | India | 522 | AC 44099 | - | - | India |
| 208 | ARC 6630 | Land race | Assam | India | 523 | AC 44100 | - | - | India |
| 209 | ARC 6631 | Land race | Assam | India | 524 | AC 44102 | - | - | India |
| 210 | ARC 6647^🞺^ | Land race | Assam | India | 525 | AC 44103 | - | - | India |
| 211 | ARC 6648 | Land race | Assam | India | 526 | AC 44115 | - | - | India |
| 212 | ARC 7008 | Land race | Assam | India | 527 | AC 44116 | - | - | India |
| 213 | ARC 7009 | Land race | Assam | India | 528 | AC 44117 | - | - | India |
| 214 | ARC 7024 | Land race | Assam | India | 529 | AC 39052 | *Tropical Japonica* | IRRI | Philippines |
| 215 | ARC 7028^🞺^ | Land race | Assam | India | 530 | IR 20 | Improved variety | IRRI | Philippines |
| 216 | ARC 7029 | Land race | Assam | India | 531 | AC 35119 | - | - | India |
| 217 | ARC 7032 | Land race | Assam | India | 532 | AC 44085 | Improved variety | - | India |
| 218 | ARC 7038 | Land race | Assam | India | 533 | MTU 1010 | Improved variety | AP | India |
| 219 | ARC 7039 | Land race | Assam | India | 534 | AC 43987 | - | - | India |
| 220 | ARC 7044 | Land race | Assam | India | 535 | CR 143-2-2 | Improved variety | Odisha | India |
| 221 | ARC 7050 | Land race | Assam | India | 536 | AG 1 | Improved variety | NRRI, Odisha | India |
| 222 | ARC 7054 | Land race | Assam | India | 537 | AG 2 | Improved variety | NRRI, Odisha | India |
| 223 | ARC 7071 | Land race | Assam | India | 538 | AG 3^🞺^ | Improved variety | NRRI, Odisha | India |
| 224 | ARC 7074 | Land race | Assam | India | 539 | AG 4 | Improved variety | NRRI, Odisha | India |
| 225 | ARC 7075 | Land race | Assam | India | 540 | AG 5 | Improved variety | NRRI, Odisha | India |
| 226 | ARC 7076 | Land race | Assam | India | 541 | AG 6 | Improved variety | NRRI, Odisha | India |
| 227 | ARC 7080 | Land race | Assam | India | 542 | AG 9 | Improved variety | NRRI, Odisha | India |
| 228 | ARC 7083 | Land race | Assam | India | 543 | AG 10 | Improved variety | NRRI, Odisha | India |
| 229 | ARC 7084 | Land race | Assam | India | 544 | AG 11 | Improved variety | NRRI, Odisha | India |
| 230 | ARC 7085 | Land race | Assam | India | 545 | AG 12^🞺^ | Improved variety | NRRI, Odisha | India |
| 231 | ARC 7086 | Land race | Assam | India | 546 | AG 13 | Improved variety | NRRI, Odisha | India |
| 232 | ARC 7093 | Land race | Assam | India | 547 | AG 14 | Improved variety | NRRI, Odisha | India |
| 233 | ARC 7094 | Land race | Assam | India | 548 | AG 15 | Improved variety | NRRI, Odisha | India |
| 234 | ARC 7104 | Land race | Assam | India | 549 | AG 16 | Improved variety | NRRI, Odisha | India |
| 235 | ARC 7105 | Land race | Assam | India | 550 | AG 17 | Improved variety | NRRI, Odisha | India |
| 236 | ARC 7106^🞺^ | Land race | Assam | India | 551 | AG 18 | Improved variety | NRRI, Odisha | India |
| 237 | ARC 7107 | Land race | Assam | India | 552 | AG 19 | Improved variety | NRRI, Odisha | India |
| 238 | ARC 7109 | Land race | Assam | India | 553 | AG 20 | Improved variety | NRRI, Odisha | India |
| 239 | ARC 7110 | Land race | Assam | India | 554 | AG 21 | Improved variety | NRRI, Odisha | India |
| 240 | ARC 7118 | Land race | Assam | India | 555 | AG 22 | Improved variety | NRRI, Odisha | India |
| 241 | ARC 7119 | Land race | Assam | India | 556 | AG 23 | Improved variety | NRRI, Odisha | India |
| 242 | ARC 7120 | Land race | Assam | India | 557 | AG 24 | Improved variety | NRRI, Odisha | India |
| 243 | ARC 7124 | Land race | Assam | India | 558 | AG 25 | Improved variety | NRRI, Odisha | India |
| 244 | ARC 7126 | Land race | Assam | India | 559 | AG 26 | Improved variety | NRRI, Odisha | India |
| 245 | ARC 7130 | Land race | Assam | India | 560 | AG 27 | Improved variety | NRRI, Odisha | India |
| 246 | ARC 7133 | Land race | Assam | India | 561 | AG 28 | Improved variety | NRRI, Odisha | India |
| 247 | ARC 7134 | Land race | Assam | India | 562 | AG 29 | Improved variety | NRRI, Odisha | India |
| 248 | ARC 7147 | Land race | Assam | India | 563 | AG 30 | Improved variety | NRRI, Odisha | India |
| 249 | ARC 7150 | Land race | Assam | India | 564 | AG 31 | Improved variety | NRRI, Odisha | India |
| 250 | ARC 7204 | Land race | Assam | India | 565 | AG 32 | Improved variety | NRRI, Odisha | India |
| 251 | ARC 7210 | Land race | Assam | India | 566 | AG 33 | Improved variety | NRRI, Odisha | India |
| 252 | ARC 7211 | Land race | Assam | India | 567 | AG 34 | Improved variety | NRRI, Odisha | India |
| 253 | ARC 7218 | Land race | Assam | India | 568 | AG 35 | Improved variety | NRRI, Odisha | India |
| 254 | ARC 7219 | Land race | Assam | India | 569 | AG 36 | Improved variety | NRRI, Odisha | India |
| 255 | ARC 7220 | Land race | Assam | India | 570 | AG 37 | Improved variety | NRRI, Odisha | India |
| 256 | ARC 7225 | Land race | Assam | India | 571 | AG 38 | Improved variety | NRRI, Odisha | India |
| 257 | ARC 7234 | Land race | Assam | India | 572 | AG 39 | Improved variety | NRRI, Odisha | India |
| 258 | ARC 7235 | Land race | Assam | India | 573 | AG 40 | Improved variety | NRRI, Odisha | India |
| 259 | ARC 7243 | Land race | Assam | India | 574 | AG 41 | Improved variety | NRRI, Odisha | India |
| 260 | ARC 7244 | Land race | Assam | India | 575 | AG 42 | Improved variety | NRRI, Odisha | India |
| 261 | ARC 7248^🞺^ | Land race | Assam | India | 576 | AG 44 | Improved variety | NRRI, Odisha | India |
| 262 | ARC 7250 | Land race | Assam | India | 577 | AG 45 | Improved variety | NRRI, Odisha | India |
| 263 | ARC 7255 | Land race | Assam | India | 578 | AG 46 | Improved variety | NRRI, Odisha | India |
| 264 | ARC 7259 | Land race | Assam | India | 579 | AG 47 | Improved variety | NRRI, Odisha | India |
| 265 | ARC 7263 | Land race | Assam | India | 580 | AG 48 | Improved variety | NRRI, Odisha | India |
| 266 | ARC 7268 | Land race | Assam | India | 581 | AG 49 | Improved variety | NRRI, Odisha | India |
| 267 | ARC 7269 | Land race | Assam | India | 582 | AG 50 | Improved variety | NRRI, Odisha | India |
| 268 | ARC 7270 | Land race | Assam | India | 583 | AG 51 | Improved variety | NRRI, Odisha | India |
| 269 | ARC 7271 | Land race | Assam | India | 584 | AG 52 | Improved variety | NRRI, Odisha | India |
| 270 | ARC 7275 | Land race | Assam | India | 585 | AG 53 | Improved variety | NRRI, Odisha | India |
| 271 | ARC 7279 | Land race | Assam | India | 586 | AG 54 | Improved variety | NRRI, Odisha | India |
| 272 | ARC 7282 | Land race | Assam | India | 587 | AG 55 | Improved variety | NRRI, Odisha | India |
| 273 | ARC 7283 | Land race | Assam | India | 588 | AG 56 | Improved variety | NRRI, Odisha | India |
| 274 | ARC 7284 | Land race | Assam | India | 589 | AG 57 | Improved variety | NRRI, Odisha | India |
| 275 | ARC 7308 | Land race | Assam | India | 590 | AG 58 | Improved variety | NRRI, Odisha | India |
| 276 | ARC 7312 | Land race | Assam | India | 591 | AG 59 | Improved variety | NRRI, Odisha | India |
| 277 | ARC 10691 | Land race | Assam | India | 592 | AG 60 | Improved variety | NRRI, Odisha | India |
| 278 | ARC 7317 | Land race | Assam | India | 593 | AG 61^🞺^ | Improved variety | NRRI, Odisha | India |
| 279 | ARC 7318 | Land race | Assam | India | 594 | AG 62 | Improved variety | NRRI, Odisha | India |
| 280 | ARC 7320 | Land race | Assam | India | 595 | AG 63 | Improved variety | NRRI, Odisha | India |
| 281 | ARC 7322 | Land race | Assam | India | 596 | AG 64 | Improved variety | NRRI, Odisha | India |
| 282 | ARC 7323 | Land race | Assam | India | 597 | AG 65 | Improved variety | NRRI, Odisha | India |
| 283 | ARC 7328 | Land race | Assam | India | 598 | AG 66 | Improved variety | NRRI, Odisha | India |
| 284 | ARC 7329 | Land race | Assam | India | 599 | AG 67 | Improved variety | NRRI, Odisha | India |
| 285 | ARC 7335 | Land race | Assam | India | 600 | AG 68 | Improved variety | NRRI, Odisha | India |
| 286 | ARC 7336^🞺^ | Land race | Assam | India | 601 | AG 69 | Improved variety | NRRI, Odisha | India |
| 287 | ARC 7339 | Land race | Assam | India | 602 | AG 70 | Improved variety | NRRI, Odisha | India |
| 288 | ARC 7341 | Land race | Assam | India | 603 | AG 71 | Improved variety | NRRI, Odisha | India |
| 289 | ARC 7342 | Land race | Assam | India | 604 | AG 72 | Improved variety | NRRI, Odisha | India |
| 290 | ARC 7343 | Land race | Assam | India | 605 | AG 73 | Improved variety | NRRI, Odisha | India |
| 291 | ARC 7408 | Land race | Assam | India | 606 | AG 74 | Improved variety | NRRI, Odisha | India |
| 292 | ARC 7410 | Land race | Assam | India | 607 | AG 76 | Improved variety | NRRI, Odisha | India |
| 293 | ARC 7412 | Land race | Assam | India | 608 | AG 77 | Improved variety | NRRI, Odisha | India |
| 294 | ARC 7414 | Land race | Assam | India | 609 | AG 79 | Improved variety | NRRI, Odisha | India |
| 295 | ARC 7415 | Land race | Assam | India | 610 | AG 80 | Improved variety | NRRI, Odisha | India |
| 296 | ARC 7416 | Land race | Assam | India | 611 | IC 301206 | Land race | - | India |
| 297 | ARC 7432 | Land race | Assam | India | 612 | AC43696 | Improved variety | Tamil Nadu | India |
| 298 | ARC 10882^🞺^ | Land race | Assam | India | 613 | AC43712^🞺^ | Improved variety | Tamil Nadu | India |
| 299 | ARC 10884 | Land race | Assam | India | 614 | AC 43847^🞺^ | Land race | Tamil Nadu | India |
| 300 | ARC 10902 | Land race | Assam | India | 615 | AC 36483 | Improved variety | IARI, New Delhi | India |
| 301 | ARC 10913 | Land race | Assam | India | 616 | AC 36691^🞺^ | Land race | Tamil Nadu | India |
| 302 | ARC 10922 | Land race | Assam | India | 617 | SR26B^🞺^ | Improved variety | NRRI, Odisha | India |
| 303 | ARC 10925 | Land race | Assam | India | 618 | Varshadhan^🞺^ | Improved variety | NRRI, Odisha | India |
| 304 | ARC 10926 | Land race | Assam | India | 619 | CR Dhan 201 | Improved variety | NRRI, Odisha | India |
| 305 | ARC 10927 | Land race | Assam | India | 620 | CR Dhan 202 | Improved variety | NRRI, Odisha | India |
| 306 | ARC 10934 | Land race | Assam | India | 621 | CR Dhan 204 | Improved variety | NRRI, Odisha | India |
| 307 | ARC 10937^🞺^ | Land race | Assam | India | 622 | ARC 10063^🞺^ | Land race | Assam | India |
| 308 | ARC 10940 | Land race | Assam | India | 623 | ARC 10075^🞺^ | Land race | Assam | India |
| 309 | AC 41647 | Land race | Bihar | India | 624 | R-255^🞺^ | Improved variety | NRRI, Odisha | India |
| 310 | AC 41620 (A) | Land race | Bihar | India | 625 | ARC 5999 | Land race | Assam | India |
| 311 | ARC 10944^🞺^ | Land race | Assam | India | 626 | ARC 11063^🞺^ | Land race | Assam | India |
| 312 | ARC 10946 | Land race | Assam | India | 627 | ARC 11133^🞺^ | Land race | Assam | India |
| 313 | ARC 10954^🞺^ | Land race | Assam | India | 628 | ARC 11296^🞺^ | Land race | Assam | India |
| 314 | ARC 10957^🞺^ | Land race | Assam | India | 629 | AC 35740^🞺^ | Land race | Assam | India |
| 315 | ARC 10958 | Land race | Assam | India | 630 | KB 46^🞺^ | Improved variety | NRRI, Odisha | India |

^[🞺-Selected 96 mini-core rice accessions for molecular profiling with early seedling vigour trait linked microsatellite markers for diversity, structure and trait-association studies]^
